# In Vitro Evaluation of Five Antimicrobial Peptides against the Plant Pathogen *Erwinia amylovora*

**DOI:** 10.3390/biom11040554

**Published:** 2021-04-09

**Authors:** Rafael J. Mendes, Laura Regalado, João P. Luz, Natália Tassi, Cátia Teixeira, Paula Gomes, Fernando Tavares, Conceição Santos

**Affiliations:** 1 Faculty of Sciences, University of Porto, 4169-007 Porto, Portugal; up201604469@edu.fc.up.pt (L.R.); catia.teixeira@fc.up.pt (C.T.); pgomes@fc.up.pt (P.G.); ftavares@fc.up.pt (F.T.); csantos@fc.up.pt (C.S.); 2 LAQV-REQUIMTE, Biology Department, Faculty of Sciences, University of Porto, 4169-007 Porto, Portugal; 3 CITAB—Centre for the Research and Technology of Agro-Environmental and Biological Sciences, University of Trás-os-Montes e Alto Douro, 5000-801 Vila Real, Portugal; 4 CIBIO—Research Centre in Biodiversity and Genetic Resources, InBIO, Associated Laboratory, Faculty of Sciences, University of Porto, 4485-661 Vairão, Portugal; 5 LAQV-REQUIMTE, Department of Chemistry and Biochemistry, Faculty of Sciences, University of Porto, 4169-007 Porto, Portugal; natalia.tcpf@fc.up.pt; 6 QRural, Polytechnic Institute of Castelo Branco, School of Agriculture, 6000-084 Castelo Branco, Portugal; j.p.luz@ipcb.pt

**Keywords:** antimicrobial activity, CA-M, Dhvar-5, D4E1, fire blight, flow cytometry, membrane permeabilization, RW-BP100, 3.1

## Abstract

Fire blight is a major pome fruit trees disease that is caused by the quarantine phytopathogenic *Erwinia amylovora*, leading to major losses, namely, in pear and apple productions. Nevertheless, no effective sustainable control treatments and measures have yet been disclosed. In that regard, antimicrobial peptides (AMPs) have been proposed as an alternative biomolecule against pathogens but some of those AMPs have yet to be tested against *E. amylovora*. In this study, the potential of five AMPs (RW-BP100, CA-M, 3.1, D4E1, and Dhvar-5) together with BP100, were assessed to control *E. amylovora*. Antibiograms, minimal inhibitory, and bactericidal concentrations (minimal inhibitory concentration (MIC) and minimal bactericidal concentration (MBC), growth and IC_50_ were determined and membrane permeabilization capacity was evaluated by flow cytometry analysis and colony-forming units (CFUs) plate counting. For the tested AMPs, the higher inhibitory and bactericidal capacity was observed for RW-BP100 and CA-M (5 and 5–8 µM, respectively for both MIC and MBC), whilst for IC_50_ RW-BP100 presented higher efficiency (2.8 to 3.5 µM). Growth curves for the first concentrations bellow MIC showed that these AMPs delayed *E. amylovora* growth. Flow cytometry disclosed faster membrane permeabilization for CA-M. These results highlight the potential of RW-BP100 and CA-M AMPs as sustainable control measures against *E. amylovora*.

## 1. Introduction

Fire blight is a destructive disease that affects primarily some chain-of-value pome fruit trees of the Rosaceae family, namely, pear (*Pyrus communis* L.), apple (*Malus domestica* Borkh.), loquats (*Eriobotrya japonica* (Thunb.) Lindl.), quince (*Cydonia oblonga* Mill.), and other ornamental species [1,2]. This disease leads to production losses, being a major concern for pome fruit producers around the world, with great economic impact where its present [3]. The etiological agent responsible for this disease is the quarantine bacterium *Erwinia amylovora* (Burril) Winslow et al., a Gram-negative of the Erwiniaceae family, which has been considered as one of the top ten plant pathogenic bacteria [4,5]. This pathogen was first reported in North America, but is currently spread to Europe (United Kingdom, Spain, France, Italy, Hungary, and Portugal), South Korea, New Zealand, and the Middle East [6,7,8,9,10,11,12,13,14].

One of the main problems of fire blight is the lack of effective control measures to stop its high destructive effects in plants and its dissemination [15,16]. Current methods rely mostly on preventive measures, with cultural control practices, like, avoidance of production areas favorable for disease development, proper fertilization, and irrigation, shortening of the blooming period, and pruning or decimation of infected trees [16,17]. Regarding the chemical controls available, the use of copper-based compounds has led to emerging phytotoxicity associated with high doses of copper in the soil, and acquisition of resistance by bacteria [18,19]. Antibiotics, like streptomycin and oxytetracycline, although reasonably effective, present serious limitations, namely, the lack of efficacy at lower doses, high production cost and phytotoxicity, which associated with the emerging antibiotic resistance, has led many regions (e.g., European Union) to ban their use, although they are still allowed in regions like North America and others [20,21]. These limitations in controlling fire blight urge for new, safe, and efficient control strategies. In response to this, one of the most studied fields in the last years is the identification of bacteriophages and antagonistic bacteria [22,23,24], and the application of essential oils [25,26]. Moreover, antimicrobial peptides (AMPs) present highly promising outcomes [27,28,29,30,31].

AMPs can be found in diverse organisms, such as insects, mammals including humans, reptiles, amphibians, and plants, being components of their immune/defense system [20,32,33]. Most AMPs are short chained cationic and amphipathic amino acids (aa), adopting α-helix, β-sheet, or loop conformations [32,33,34,35]. They possess a broad spectrum of action against numerous microorganisms, including fungi and bacteria, due to their capacity to interact with microbial cell membranes, which confers them an antimicrobial activity mainly, but often not exclusively, due to membrane permeation or disruption (e.g., formation of pores or micelles, respectively) [32,33,34,36]. AMPs can also interact with intracellular targets, including ribosomes, or nucleic acids after traversing the cell membrane, which leads to inhibition of DNA transcription, mRNA synthesis, protein synthesis, and lead to formation of reactive oxygen species (ROS) [32,33]. One of the advantages of the AMPs is that generally they do not possess specific enzyme targets like antibiotics do, which propels AMPs less likely to draw resistance from microbial mutations [31,33].

Several AMPs have been reported active against several Gram-negative plant pathogenic bacteria, such as *E. amylovora*, *Pseudomonas syringae*, *Xanthomonas vesicatoria* [37], *Erwinia carotovora* subsp. *carotovora*, *Erwinia carotovora* subsp. *atroseptica* [38,39], *Xylella fastidiosa* [40], *Xanthomonas oryzae*, *Xanthomonas campestris* [41], and *Pseudomonas syringae* pv. *actinideae* [42], all with an outer-membrane containing lipopolysaccharide (LPS) that serves as permeability membrane against chemical compounds [43]. One such AMPs is the linear undecapeptide BP100 (KKLFKKILKYL-NH2), with bioactivity against *E. amylovora*. BP100 has high antimicrobial activity, inducing low hemolysis and phytotoxicity [29,44,45,46]. Nevertheless, BP100 has limited activity against Gram-negative bacteria, and to surpass that limitation an analogue was developed, namely, RW-BP100 (RRLFRRILRWL-NH2), which demonstrated improved efficacy against Gram-negative bacteria such as *Escherichia coli*, *Klebsiella pneumoniae,* and *Pseudomonas aeruginosa*, whilst demonstrating efficacy in some Gram-positive bacteria [47]. Other AMP reported as effective against Gram-negative bacteria include the cecropin A-melittin (CA-M) hybrid (KWKLFKKIGAVLKVL-NH2) [48,49] and peptide 3.1 (KKLLKWLLKLL-NH2), an LK peptide studied in the last years, with promising results against e.g., *Shigella dysenteriae*, *K. pneumoniae*, and *E. coli* [50,51]. Another AMP with demonstrated efficacy against Gram-negative *Pseudomonas syringae* pv. *tabaci* and *Xanthomonas campestris* pv. *malvacearum* bacteria is D4E1 (FKLRAKIKVRLRAKIKL-NH2) [52]. Relevantly, none of these promising AMPs, RW-BP100, CA-M, 3.1, or D4E1, have ever been tested against *E. amylovora*, which compelled us to fill this gap. Indeed, identification of valuable AMPs to fight plant pathogens and tackle crop-relevant infectious diseases is still underway.

Hence, the effect of BP100, RW-BP100, CA-M, 3.1, and D4E1 was evaluated in this work, at different doses and on a collection of *E. amylovora*, to assess the range of bioactivity of these AMPs in this bacterium. For comparison, we have also included the AMP Dhvar-5 (LLLFLLKKRKKRKY-NH2), widely reported to possess potent action against Gram-positive bacteria like *Staphylococcus aureus* [53] and which was not tested against *E. amylovora* before either. Dose effects were compared by performing concentration tests and assessing the integrity of cell membrane and cell viability, to disclose the most effective AMPs.

## 2. Materials and Methods

### 2.1. Peptide Synthesis

BP100, RW-BP100, CA-M, D4E1, 3.1 and Dhavar-5 peptides (Table 1) were assembled following an orthogonal Fmoc/tBu scheme [54], on a Rink-amide-MBHA-derivatized matrix of copoly (styrene/1% divinylbenzene), 100–200 mesh, 0.36 mmol·g^−1^ functionalization (NovaBiochem, Merck KGaA, Darmstadt, Germany). Peptide synthesis was performed on an automated Symphony X synthesizer from Gyros Protein Technologies (Tucson, AZ, USA), at the Laboratory of Peptide and Peptide-Nucleic Acid Synthesis of the Faculty of Sciences of the University of Porto (POP-UP). All peptides presented a purity degree (>98%) that was quantitated by analytical reverse-phase high performance liquid chromatography (RP-HPLC) using a Hitachi-Merck LaChrom Elite system equipped with a quaternary pump, a thermostated automated sampler, and a diode-array detector (DAD). Analyses were performed with a reverse-phase C18 column (150 × 4.6 mm ID and 5 μm pore size, Merck) at a 1 mL/min flow rate using a 1–100% of solvent B (ACN) in solvent A, for 30 min, with detection at 220 nm. An LCQ-DecaXP LC-MS system from ThermoFinnigan, equipped with both a DAD detector and an electrospray ionization-ion trap mass spectrometer (ESI/IT MS) was used to confirm peptide identity. RP-HPLC chromatograms and ESI-IT MS spectra of synthesized peptides are provided as Supplementary Information (Figures S1–S12).

### 2.2. Bacterial Strains

Thirty-six *E. amylovora* strains, collected in pear and apple orchards from the North and Centre of Portugal between 2010 and 2017, were evaluated (Table 2). Type strain LMG 2024 was used as a reference. Bacterial strains were preserved at −80 °C in 30% glycerol and 70% King’s B (KB) medium. Otherwise stated, strains were cultured in KB medium at 28 °C.

### 2.3. Antibiogram Assay

The antibiogram assay was applied to screen the effects of the six AMPs against *E. amylovora*. The 37 bacterial strains were grown overnight in Mueller Hinton (MH) Broth (Liofilchem, Téramo, Italy) at 25 °C and 180 rpm. A bacterial suspension was adjusted to 0.1 at an optical density at 600 nm (OD_600_), which corresponds to approximately between 1 × 10^8^ to 1 × 10^9^ CFU·mL^−1^, and 1 mL of the bacterial suspension was grown on MH agar medium (Liofilchem, Téramo, Italy). Subsequently, 1 µL of eight concentrations (0.4, 1.6, 6.2, 25, 50, 100, 150, and 200 µM) of each AMP were applied directly in the Petri dish. After 24 h of incubation at 25 °C, results were recorded using the Gel Doc XR+ (Bio-Rad Laboratories, Hercules, CA, USA) to evaluate the inhibition halo. Results were considered positive (+) when halo formation occurred, and negative (−) when there was no observable halo. Each experiment was repeated three times.

### 2.4. Antimicrobial Activity of AMPs

To assess the antimicrobial activity of the AMPs, four parameters were evaluated, namely, the minimal inhibitory concentration (MIC), the minimal bactericidal concentration (MBC), the half-maximal inhibitory concentration (IC_50_), and the growth during 24 h. After analyzing the results of the antibiogram assay, only the three most effective AMPs against *E. amylovora* were further tested, namely, BP100, RW-BP100, and CA-M, and their concentrations were adjusted to 1.6, 3.4, 5, 8, 12, 20, 30, 70, and 100 µM. *E. amylovora* strains used were the most representative of the collection, namely, Ea 230, Ea 320, Ea 390, Ea 490, Ea 630, Ea 680, and Ea 820, and the type strain LMG 2024 (Table 1), which corresponded to strains that were collected from different years, different hosts from several orchards and with different virulence levels (data not shown). For the MIC assay, the bacterial strains were grown overnight in 2× MH broth at 25 °C and 180 rpm and then adjusted to an OD_600_ of 0.1. The bacterial suspension (75 µL) was applied in a 96-well titration plate, to which the nine concentrations of each AMP were previously added (75 µL each), in order to obtain a final concentration of 1:1. During a period of 24 h, the titration plate was incubated at 25 °C in constant shaking, with hourly absorbance reading at 600 nm, in the Multiskan™ GO (Thermo Fisher Scientific, Waltham, MA, USA). Results were obtained after 24 h when inhibition of growth was visibly detected. The positive control was chlortetracycline (50 µM) and the negative control was H_2_O. Immediately afterwards, 10 µL from the bacterial suspensions of each well were grown on KB at 25 °C during 24 h. MBC concentration was obtained when there was no visible colony growth. Since equal values of MIC and MBC could be obtained for the same AMP in different strains, IC_50_ values were determined through non-linear fitting, resorting to GraphPad Prism 8 software (GraphPad Software, San Diego, CA, USA), and growth curves were assessed for the concentrations below the MIC value of each strain for each AMP. Each experiment was repeated three times.

### 2.5. Evaluation of AMPs Membrane Permeabilization through Flow Cytometry

For in vitro assessment of the AMPs in cell viability, flow cytometry (FC) was used. *E. amylovora* type strain LMG 2024 was grown overnight in KB broth at 25 °C and 180 rpm, later the bacterial culture was centrifuged for 5 min at 2500 rpm. Pellet was resuspended in PBS (10 mM, pH 7.2), and OD_600_ was adjusted to 0.1. The concentrations tested for BP100, RW-BP100, and CA-M were 5 and 8 µM, which correspond to the MIC values obtained previously. An aliquot of 50 µL of bacteria with the respective AMP was stained with propidium iodide (PI) in a final concentration of 1 mg·mL^−1^. Fluorescence intensities were recorded at t_0_, t_10_, t_30_, t_60_, and t_120_ min. Flow cytometry was performed on a BD Accuri™ C6 Plus (BD Bioscience, Franklin Lakes, NJ, USA). Data were collected for a total of 20,000 events and analyzed by gating using flow cytometry software C6 Plus Analysis (BD Bioscience, Franklin Lakes, NJ, USA). Viability was inversely proportional to PI fluorescence level. Isopropyl alcohol 23% was used as a positive control. Each experiment was repeated three times.

### 2.6. Assessment of Colony Forming Units (CFUs)

The colony-forming units (CFU) method was applied to assess the number of viable cells after treatment with BP100, RW-BP100, and CA-M in the FC assay. Briefly, after 120 min of exposure of the *E. amylovora* type strain LMG 2024 to each AMP at different concentrations (5 and 8 µM), a tenfold dilution series was applied. After that, 10 µL of each bacterial dilution were applied directly on KB medium in triplicate, followed by incubation at 28 °C for 24 h. Photographs were obtained for each treatment for each AMP, and the number of colonies obtained was counted to assess CFU. Each experiment was repeated three times.

### 2.7. Statistical Analysis

Comparisons between the treatments for IC_50_, and CFU were analyzed through One-way Anova, whilst FC was analyzed through Two-way Anova using GraphPad Prism 8 for Windows (GraphPad Software, San Diego, CA, USA). Results were considered statistically different when *p* < 0.05.

## 3. Results

To assess the efficacy of six AMPs (BP100, RW-BP100, CA-M, 3.1, D4E1, and Dhvar-5) against *E. amylovora*, a diverse collection of strains of this pathogen was tested for several endpoints, regarding their susceptibility and viability.

### 3.1. Peptide Synthesis

All peptides listed in Table 1 were synthesized by standard solid-phase peptide synthesis (SPPS) protocols based on the Fmoc/tBu orthogonal protection scheme [54]. The peptides were purified with high purity (≥98%), according to reverse-phase high performance liquid chromatography (RP-HPLC) analysis, and their expected molecular weights (MW) were confirmed by electrospray ionization-ion trap mass spectrometry (ESI-IT MS) (Figures S1–S12).

### 3.2. Antibiogram Assay

**Figure 1:**
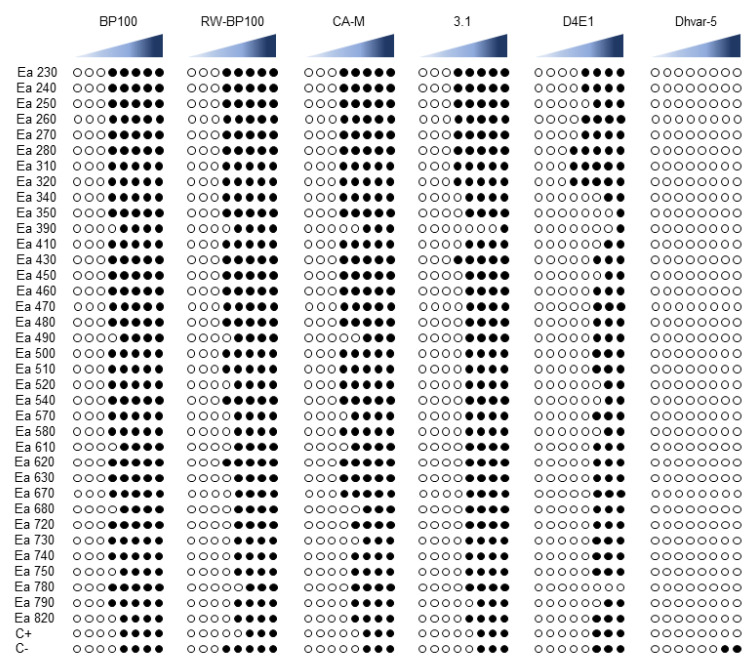
Antibiogram susceptibility to antimicrobial peptides (AMPs) BP100, RW-BP100, CA-M, 3.1, D4E1, and Dhvar-5. Each AMP was tested using eight concentrations, namely, 0.4, 1.6, 6.2, 25, 50, 100, 150, and 200 µM (increasing concentrations are represented at blue, left to right). ◯: Normal growth; ●: Inhibited growth. C+: Type strain LMG 2024.

The antibiogram assay for the six AMPs disclosed different levels of susceptibility to each peptide, with some AMPs revealing higher efficacy than others (Figure 1, Supplementary Table S1). BP100 was the most effective AMP, with 30 strains susceptible at 25 µM (83.3%), and only 6 strains susceptible at 50 µM (16.7%), with the type strain LMG 2024 presenting this same result. Regarding RW-BP100 and CA-M, both AMPs showed similar efficiency, with 20 and 24 strains being susceptible at 25 µM (55.6 and 66.7%), 15 and 8 strains susceptible at 50 µM (41.6 and 22.2%), and 1 and 4 strains susceptible at 100 µM (2.8 and 11.1%), respectively. Type strain LMG 2024 was susceptible at 100 µM for both RW-BP100 and CA-M. The strains tested, after exposure to 3.1 and D4E1 AMPs, presented lower susceptibility results, with 3.1 displaying 9 strains susceptible at 50 µM (25%), 25 strains susceptible at 100 µM (69.4%), 1 strain susceptible at 150 µM (2.8%), and 1 strain susceptible at 200 µM (2.8%), while type strain LMG 2024 presented susceptibility at 100 µM. Regarding the exposure to D4E1, 3 strains were susceptible at 25 µM (8.3%), 4 strains were susceptible at 50 µM (11.1%), 19 strains were susceptible at 100 µM (52.8%), 7 strains were susceptible at 150 µM (19.4%), 2 strains were susceptible at 200 µM (5.6%), and 1 strain did not present any susceptibility (2.8%). Type strain LMG 2024 exposed to D4E1 displayed susceptibility at 100 µM. For Dhvar-5 the strains tested showed no susceptibility.

### 3.3. Antimicrobial Activity of AMPs

After the first screening of the six AMPs activity against the 36 strains of *E. amylovora*, the three most efficient AMPs were chosen to be further analyzed against eight representative strains of that pathogen (Table 3).

**Figure 2:**
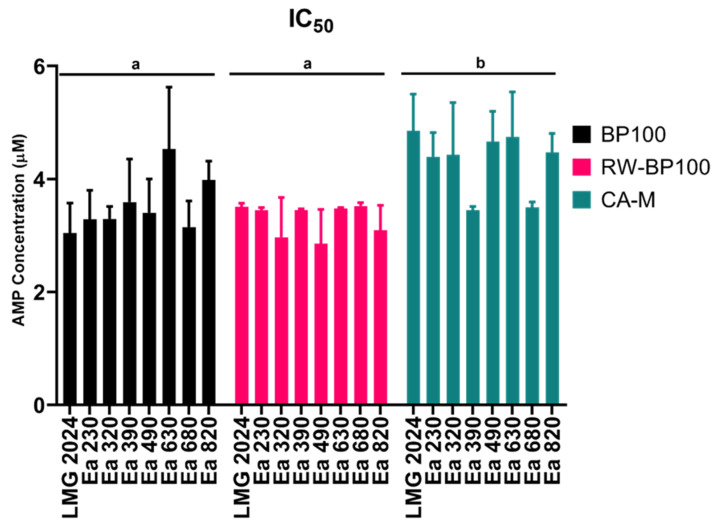
Half maximal inhibitory concentration (IC_50_) of eight *Erwinia amylovora* strains against three AMPs: BP100, RW-BP100, and CA-M. Vertical bars: mean value with standard deviation (*n* = 3). Different letters means significant differences for each AMP assay (*p* < 0.0001).

The MIC values ranged between 5 and 8 µM for both BP100 and CA-M, whilst 5 µM was obtained for RW-BP100 against all strains. The MBC values ranged between 8 and 20 µM for BP100 and between 5 and 8 µM for CA-M, while for RW-BP100 they were equal to the MIC values (5 µM). The MIC and MBC values displayed the same results for some strains, and IC_50_ allowed to further differentiate those results (Figure 2).

Although no statistically significant difference was observed between the strains in each AMP, different susceptibility for each AMP was detected among them regarding the tested strains (*p* < 0.05), namely, between BP100 and CA-M, and RW-BP100 and CA-M, with BP100 and RW-BP100 presenting generally lower IC_50_ values than CA-M. In addition to IC_50_, growth curves were analyzed for every strain for each AMP regarding the concentrations below their respective MIC (Supplementary Figures S13–S15). For BP100, every strain exposed to 1.6 µM displayed similar growth to those that were not exposed to the peptide, while the strains exposed to the concentration of 3.4 µM (LMG 2024 and Ea 320 strains), and 5 µM (remaining strains), had a considerably delayed lag phase, starting around 14–16 h after those strains either exposed to 1.6 µM or not exposed (Supplementary Figure S13). Regarding the strains exposed to RW-BP100 at 1.6 µM, growth was similar to those not exposed to the peptide; in turn, when exposed to 3.4 µM, the lag phase was delayed, but contrary to BP100, the majority started their growth around 6–8 h after the strains that were either exposed to 1.6 µM or not exposed (Supplementary Figure S14). Strains exposed to 1.6 and 3.4 µM of CA-M presented similar behavior to that of those exposed to RW-BP100, but when exposed to a concentration of 5 µM they presented the same behavior than the ones exposed to the same concentration of BP100 (Supplementary Figure S15).

### 3.4. Evaluation of AMPs Membrane Permeabilization through Flow Cytometry

**Figure 3:**
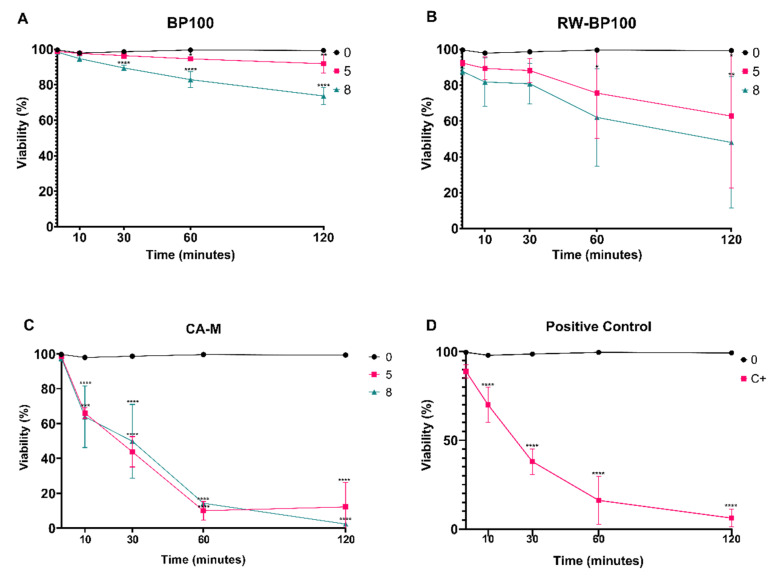
Viability of *Erwinia amylovora* strain LMG 2024 after exposure to increasing concentrations of AMPs during 120 min. (**A**) BP100; (**B**) RW-BP100; (**C**) CA-M; and (**D**) Isopropyl alcohol 23%. Viability was inversely proportional to propidium iodide (PI) fluorescence level in each time point. Vertical bars: mean value with standard deviation (*n* = 3); *, **, ***, and **** refer to the statistical significances for differences in each time point of the analysis with *p* < 0.05, *p* < 0.01, *p* < 0.001, and *p* < 0.0001, respectively.

To disclose the peptide-induced bacterial membrane disruption, flow cytometry was applied resorting to a dye that intercalates with nucleic acids when membrane permeabilization occurs, in order to quantify the loss of membrane integrity caused by the AMPs. The results obtained regarding cell viability were inversely proportional to the fluorescence level obtained for each concentration during a period of time (Figure 3).

BP100 started to show statistically significant values (*p* < 0.0001) in the decrease of cell viability at 30-min exposure at the concentration of 8 µM (89.5%). After 60 and 120 min, the same decrease of cell viability was observed (82.9 and 73.7%, respectively) (*p* < 0.0001), while for the exposure at 5 µM the decrease of cell viability was statistically significant after 60 and 120 min (94.6 and 91.9%, respectively) (*p* < 0.05 and 0.001, respectively) (Figure 3A). Regarding RW-BP100, cell viability decreased (*p* < 0.05) at 5 and 8 µM after 120-min exposure (62.8 and 48.1% respectively), and after 60-min exposure at 8 µM (62%) (Figure 3B). CA-M was the most efficient AMP, with a significant (*p* < 0.0001) reduction in cell viability throughout all time points for every concentration tested, except at time of exposure (t_0_) (Figure 3C), with expressive decrease at 60 and 120 min (below 15%). Positive control displayed a statistically significant reduction (*p* < 0.0001) of cell viability similar to CA-M (Figure 3D).

### 3.5. Assessment of Colony Forming Units (CFUs)

**Figure 4:**
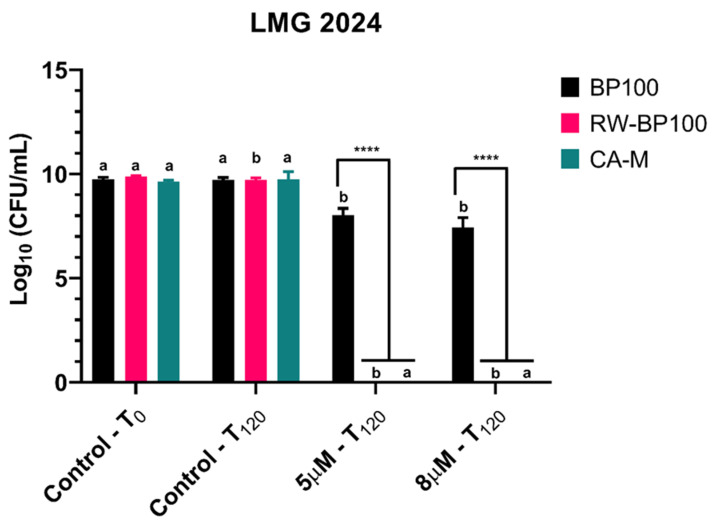
Number of viable cells of *Erwinia amylovora* strain LMG 2024 after treatment with different concentrations of AMPs for flow cytometry (FC) assay. Vertical bars: mean value with standard deviation (*n* = 3); **** refer to the statistical significances for differences in each individual concentration of the analysis with *p* < 0.0001. Different letters indicate statistical differences between treatments (*p* < 0.0001).

In order to further disclose and confirm the FC results, CFU plate counting was employed for every condition after a 120-min exposure to AMPs (Figure 4). For BP100, the 5 and 8 µM concentrations displayed a statistically significant decrease (*p* < 0.05) of CFU in comparison with the control (5.5 × 10^9^ CFU·mL^−1^ and 4 × 10^7^ CFU·mL^−1^, respectively). RW-BP100 led to a statistically significant reduction (*p* < 0.05) in CFU in the control condition after 120 min since the start of the assay and no recovery of the bacterium was observed for every concentration tested (*p* < 0.05). For CA-M, no recovery after 24 h of incubation in the plates was observed for every concentration tested (*p* < 0.05). Furthermore, at the concentrations of 5 and 8 µM, BP100 presented statistically significant difference (*p* < 0.0001) when compared with RW-BP100 and CA-M, due to recovering cells (Figure 4).

## 4. Discussion

AMPs have taken the spotlight, mainly in the last 15 years, as green bio-and eco-friendly compounds with potential to be used in agriculture to control phytopathogenic bacteria and fungi, complementing or replacing the existing agrochemicals. Nevertheless, some AMPs that have proven efficacy against a batch of plant pathogenic bacteria have not yet been tested in *E. amylovora*. Additionally, most of the studies with AMPs relied on one or very few strains of each pathogen, not providing a representative information of that population’s behavior to the AMPs [33,44,46,55].

This study, besides the previously tested BP100, assessed for the first time the effect of five AMPs, using a wide and diverse number of wild and reference strains, thus providing a more representative information of the behavior of *E. amylovora* populations to the AMPs, with D4E1 being the only one previously tested against other phytopathogenic bacteria [52].

The antibiogram screened the effect of the six AMPs on 36 strains, disclosing which AMP were more efficient, and provided average values which could be considered representative of the bacteria population. This first screening showed that the AMPs 3.1, D4E1, and Dhvar-5 were less effective than BP100, RW-BP100, and CA-M. Regarding the AMPs to which *E. amylovora* was more susceptible, the results with the 36 field strains tested show that the effect of the peptides is virtually similar for both the field and reference strains, with 83.3, 55.6, and 66.7% of the strains displaying the same susceptibility for BP100, RW-BP100, and CA-M, respectively, for the lower concentration that created a halo.

Previous works described that D4E1 was highly efficient against *P. syringae* pv. *tabaci* and *X. campestris* pv. *malvacearum*, with MIC values of 2.25 and 1.25 µM [52], while peptide 3.1 showed MIC values of 3.1 µM against *K. pneumoniae*, but at the same time demonstrated a MIC value of 25 µM against *Pseudomonas aeruginosa* [50]. Although Dhvar-5 has shown potential in inhibiting some Gram-positive bacteria [56], its mode of action, previously hypothesized to differ from that of the other five tested AMPs, did not present efficacy against *E. amylovora*. This propels the need for further bioactivity and molecular studies for the test peptides, to infer on the molecular basis for their distinctive efficiency against Gram-negative phytopathogenic bacteria. In fact, considering that different AMPs may disrupt or permeabilize the bacterial membranes and may undergo different conformational changes upon adsorption onto those membrane [57], thus influencing their mode of action, further studies with the active peptides should be made to investigate the molecular basis of their bioactivity against *E. amylovora*.

The differential susceptibility of *E. amylovora* against the test AMPs, as compared to those of other pathogens, can be explained by the different lipid composition of its cell membrane, which will therefore influence how the AMPs interact with it [58].

Evaluation of the antibacterial activity demonstrated that the peptides RW-BP100 and CA-M presented activities like that of the reference AMP BP100, which had been already tested on *E. amylovora* [29,44,59]. These studies showed BP100 MIC values between 2.5 and 7.5 µM for *E. amylovora*, which is in line with our observations for the strains tested. Interestingly, MIC values for the three best AMPs herein tested are lower than those recently reported for other peptides [31].

The MIC values for CA-M were lower or similar to those previously obtained for the same peptide against other Gram-negative bacteria, e.g., *Acinetobacter baumannii* and *E. coli*, which disclosed MIC and MBC ranging from 4 to 64 µM and 16 to 128 µM, respectively [60]. In the study of Oddo et al. [55], RW-BP100 displayed MIC values of 8 µM against distinct strains of *P. aeruginosa* and *K. pneumoniae*, whilst a strain of *E. coli* showed MIC of 2 µM, which is respectively higher and lower than the values obtained in the present study (5 µM).

Regarding the bactericidal versus bacteriostatic effects of these AMPs, data herein reported are in accordance with what had been reported in other studies on RW-BP100, which showed lower MBC values than BP100 against Gram-negative bacteria, namely, *E. coli* and *P. aeruginosa* [47]. Furthermore, MBC values for RW-BP100 match their MIC values, disclosing an effective bactericidal effect at 5 µM, whilst BP100 displays a bacteriostatic effect at the same concentration, being bactericidal only at concentrations from 8 to 20 µM. The fact that CA-M displayed similar values of MBC and MIC, as it happened with RW-BP100, show that CA-M also presents a bactericidal effect against *E. amylovora*, in line with what was previously reported for this peptide against other Gram-negative pathogens [61]. Bactericidal compounds cause bacterial cell death, which means that their inhibitory effects remain constant since the moment they are applied. On the other hand, bacteriostatic compounds merely inhibit normal bacterial growth, which means that their inhibition effects are exerted only when the bacteria are exposed to them; hence, bacteria can restore their growth capacity once optimal conditions are resumed [44].

To further assess possible variability of the effects of the AMPs in different strains, the IC_50_ was calculated. IC_50_ values are in accordance with the MIC values obtained for each strain and for each AMP. Thus, the peptide that induced lower IC_50_ for *E. amylovora* was RW-BP100 (30% less the MIC value), with CA-M presenting the higher IC_50_ values. Interestingly, both RW-BP100 and BP100 displayed significantly different group IC_50_ values when compared to CA-M. These distinct IC_50_ values may be due to slight differences in either conformation or bacterial killing kinetics among the peptides, which can be evaluated through the growth of the bacterium.

Regarding the sub-lethal doses of the three AMPs, it was possible to disclose that these peptides induce changes in bacterial growth at doses as low as 3.4 µM, especially for BP100, being those changes more pronounced at the concentration immediately below the MIC, causing a significantly delay in the growth lag phase and therefore also in the exponential phase of *E. amylovora*. These results are in alignment with the MIC values determined for the same strains. The stimulus that occurs in some strains at lower concentrations (3.4 µM), which proved to be a non-lethal concentration, may be due to a stress that happens in the pathogen, as already reported for *Listeria monocytogenes* [62]. This serves as an indicator of the bactericidal effect of these AMPs, since above these values no growth was observed. Further studies must be applied to obtain further clarification about these factors. Although different methodologies can be used to evaluate IC_50_, our results are aligned with previous studies.

The results presented in this study further support what has been previously established for the AMPs activity in other pathogens, which may be due to the various modes of action that these peptides induce cell membrane disruption; this is related to their aa sequence, which can lead to different modes of membrane-destabilizing action (e.g., barrel stave-poration, toroidal poration, non-porating carpet model, charge-based phospholipid clustering, among others) [63,64]. These differences can also be attributed to factors not related to the AMPs, such as, the phospholipid composition and/or fluidity of the membrane, pH, temperature and ionic stress [64], thus proving the necessity to widen the research of these biomolecules against a larger panel of bacteria.

Pathogen cell viability has been evaluated before resorting to FC, to infer bacterial membrane permeability [29,65,66,67]. In the present work, FC was used to test every AMP for their membrane-permeabilizing action on *E. amylovora*, showing that increasing concentrations of the peptides cause an increase in the fluorescence signal due to PI, i.e., a dose dependent response was obtained that correlates with the extent of bacterial membrane disruption. This is in accordance with what was previously described by O’Brien-Simpson [68], and further confirm that the bactericidal effects of these AMPs are principally associated with disruption of the pathogen cell membrane, which agrees with previous studies of these AMPs [44,47,64,69].

Type strain LMG 2024 presented decreased cell viability in the presence of all BP100 concentrations tested, with the most pronounced results (decrease of 73.7%) being achieved after a 120-min exposure to 8 µM. BP100 effects on *E. amylovora* may not be too severe, allowing for its cells to recover in optimal conditions, as it was shown in the UFC plate counting. These findings suggest that CA-M has the fastest action in decreasing the total cell viability, at low concentrations (5 and 8 µM), which was corroborated by plate counting, where no bacteria grew after 24 h of incubation. At the same time, CA-M induced the higher membrane permeability at both concentrations, with cell viability decreasing below 50% after 30 min of exposure, and after 60 min it was decreased below 15% for all tested concentrations. These results demonstrate a great potential for this peptide to control *E. amylovora*. Regarding the cell viability of the strains exposed to RW-BP100, once again the higher concentrations led to higher viability loss. This demonstrates a positive linear relation between AMPs concentration and reduction of cell viability.

Plate counting after 24 h showed that when *E. amylovora* was exposed to both RW-BP100 and CA-M peptides, there was no cell recovery/division, which is line with the bactericidal effect previously determined. Plate counting for RW-BP100 showed no viable cells after 24 h, and MIC and MBC values of 5 µM, being in line with the proposed mechanism of action for this AMP, i.e., bacterial cell membrane disruption [47].

Our results allow to infer that besides being dose-dependent, cell viability, and therefore, membrane disruption/permeabilization occurs along time starting from exposure to the tested AMPs, with a significant decrease after 60 min for CA-M, and 120 min for RW-BP100. This time-dependent membrane disruption is in accordance to what has been hypothesized for the mode of action of the AMPs, in which they accumulate over time on the outer membrane, gradually promoting increasing disturbance in the lipid ordering, until disruption occurs [33]. Furthermore, this confers these AMPs a fast activity against the target pathogen. Both CA-M and RW-BP100 presented improved bioactivity as compared to BP100 at the same concentrations, which could propel them as improved biomolecular compounds to control this disease. Moreover, CA-M and RW-BP100 could be used in synergy with each other, or with BP100, in order to increase antibacterial potency, as several recent studies have highlighted the benefits of synergic interactions with AMPs [29,70,71,72]. Furthermore, these results shows that both RW-BP100 and CA-M, as also BP100 possess structures that allows them to successfully interact with the LPS of *E. amylovora*, which leads to membrane permeation, as it has been proved before as a critical aspect for AMPs optimal efficiency [43].

Overall, when comparing BP100 with both CA-M and RW-BP100, the latter showed better efficiency, with lower MIC, MBC, IC_50_, FC, and UFC plate counting values. These observations, along with low hemolytic activity previously reported for CA-M [44,73,74], advances these peptides as novel suitable candidates to be applied in agriculture control measures against bacterial diseases like fire blight.

## 5. Conclusions

This work had the objective of testing for the first time five AMPs against a diverse collection of *E. amylovora*, and compare their efficiency with that of BP100 previously described as effective against the pathogen. Our findings suggest that RW-BP100 and CA-M peptides are highly active against *E. amylovora*, making them good candidates to control this pathogen, since in the antimicrobial, cell viability and cell recovery assays they demonstrated better results than BP100. Data also suggest a population reproducibility regarding *E. amylovora*. Nevertheless, future studies *in planta*, and in vitro must be applied in order to further disclose the function and efficacy of these AMPs, and how they affect the metabolism of the pathogen and interact with the bacteria membrane. Additionally, synergic studies with the combination of the most effective AMPs found here would represent a new step to the use of AMPs in agriculture.

## Figures and Tables

**Table 1:** Sequence and properties of peptides used in this work.

Peptide	Sequence	Net Charge ^1^	MW (Da) ^2^
BP100	KKLFKKILKYL-NH_2_	+6	1419.9
RW-BP100	RRLFRRILRWL-NH_2_	+6	1583.0
CA-M	KWKLFKKIGAVLKVL-NH_2_	+6	1769.2
D4E1	FKLRAKIKVRLRAKIKL-NH_2_	+9	2079.4
3.1	KKLLKWLLKLL-NH_2_	+5	1393.9
Dhvar-5	LLLFLLKKRKKRKY-NH_2_	+8	1845.3

^1^ Estimated net charge at pH 7; ^2^ MW: molecular weight. Source: Pepdraw.com accessed on 01 March 2021.

**Table 2:** Portuguese *Erwinia amylovora* strains used in this work.

Strain	Host		Isolated From	Geographic Origin	Year
	Species	Cultivar			
Ea 230	Pear	‘Rocha’	Exudate	Alcobaça	2010
Ea 240	Pear	‘Rocha’	Exudate	Alcobaça	2010
Ea 250	Pear	‘Rocha’	Branch	Alcobaça	2010
Ea 260	Pear	‘Rocha’	Branch	Alcobaça	2010
Ea 270	Pear	‘Passe Crassane’	Branch	Alcobaça	2010
Ea 280	Pear	‘Rocha’	Exudate	Alcobaça	2011
Ea 310	Pear	‘Rocha’	Exudate	Alcobaça	2011
Ea 320	Pear	‘Rocha’	Branch	Alcobaça	2011
Ea 340	Pear	‘Rocha’	Branch	Alcobaça	2011
Ea 350	Pear	‘Rocha’	Branch	Alcobaça	2011
Ea 390	Apple	‘Royal Gala’	Necrotic fruit	Alcobaça	2011
Ea 410	Apple	‘Royal Gala’	Semi-necrotic fruit	Alcobaça	2011
Ea 430	Apple	‘Royal Gala’	Semi-necrotic fruit	Alcobaça	2011
Ea 450	Pear	‘Rocha’	Exudate	Alenquer	2015
Ea 460	Pear	‘Rocha’	Exudate	Alenquer	2015
Ea 470	Pear	‘Rocha’	Exudate	Alenquer	2015
Ea 480	Pear	‘Rocha’	Exudate	Alenquer	2015
Ea 490	Pear	‘Rocha’	Branch	Alenquer	2015
Ea 500	Pear	‘Rocha’	Branch	Alenquer	2015
Ea 510	Pear	‘Rocha’	Exudate	Alenquer	2015
Ea 520	Pear	‘Rocha’	Exudate	Alenquer	2015
Ea 540	Pear	‘Carapinheira’	Branch	Caldas da Rainha	2015
Ea 570	Pear	‘Carapinheira’	Branch	Caldas da Rainha	2015
Ea 580	Pear	‘Carapinheira’	Branch	Caldas da Rainha	2015
Ea 610	Apple	‘Gala’	Branch	Cadaval	2015
Ea 620	Apple	‘Gala’	Branch	Cadaval	2015
Ea 630	Apple	‘Gala’	Branch	Cadaval	2015
Ea 670	Pear	‘Rocha’	Branch	Cadaval	2015
Ea 680	Pear	‘Rocha’	Branch	Cadaval	2015
Ea 720	Pear	‘Rocha’	Branch	Cadaval	2015
Ea 730	Pear	Unidentified	Branch	West *	2017
Ea 740	Pear	Unidentified	Branch	West *	2017
Ea 750	Pear	Unidentified	Branch	West *	2017
Ea 780	Pear	Unidentified	Branch	West *	2017
Ea 790	Pear	Unidentified	Branch	West *	2017
Ea 820	Pear	Unidentified	Branch	West *	2017

* These isolates have been isolated in the West region of Portugal, which includes the municipalities of Alcobaça, Caldas da Rainha, Alenquer and Cadaval.

**Table 3:** AMPs values of minimal inhibitory concentration (MIC), minimal bactericidal concentration (MBC) and obtained for the eight *Erwinia amylovora* strains (*n* = 3).

AMP	Strain	MIC (µM)	MBC (µM)
BP100	LMG 2024	5	8
	Ea 230	8	8
	Ea 320	5	8
	Ea 390	8	20
	Ea 490	8	12
	Ea 630	8	8
	Ea 680	8	8
	Ea 820	8	8
RW-BP100	LMG 2024	5	5
	Ea 230	5	5
	Ea 320	5	5
	Ea 390	5	5
	Ea 490	5	5
	Ea 630	5	5
	Ea 680	5	5
	Ea 820	5	5
CA-M	LMG 2024	8	8
	Ea 230	8	8
	Ea 320	8	8
	Ea 390	5	5
	Ea 490	8	8
	Ea 630	8	8
	Ea 680	5	5
	Ea 820	8	8

## Data Availability

Following the MDPI Research Data Policies, data from this paper will be available under request.

## Supplementary Materials

The following are available online at https://www.mdpi.com/article/10.3390/biom11040554/s1, Figure S1: Mass spectrum (ESI-IT, positive mode) of peptide BP100 (MW = 1419.9 Da), highlighting the quasi-molecular ion ([P+H]^+^), its sodium adduct ([P+Na]^+^, base peak), the di-protonated ([P+2H]^2+^) ion and its sodium adduct ([P+2Na]^2+^), and the tri-protonated ([P+3H]^3+^) and tetra-protonated ([P+4H]^4+^) ions of the target peptide (P), Figure S2: RP-HPLC chromatogram for peptide BP100, after purification; gradient elution from 1 to 100% ACN in 0.05% aqueous TFA at 1 mL/min flow rate, for 30 min, on a C-18 column (150 × 4.6 mm ID and 5 µm pore size); detection at 220 nm, Figure S3: Mass spectrum (ESI-IT, positive mode) of peptide RW-BP100 (MW = 1583.0 Da), highlighting the quasi-molecular ion ([P+H]^+^), the di-protonated ([P+2H]^2+^, base peak) ion and respective sodium adduct ([P+2Na]^2+^), the tri-protonated ion ([P+3H]^3+^) and its sodium and dipotassium adduct ([P+Na+2K]^3+^), and the tetra-protonated ([P+4H]^4+^) ion of the target peptide (P), Figure S4: RP-HPLC chromatogram for peptide RW-BP100, after purification; gradient elution from 1 to 100% ACN in 0.05% aqueous TFA at 1 mL/min flow rate, for 30 min, on a C-18 column (150 × 4.6 mm ID and 5 µm pore size); detection at 220 nm, Figure S5: Mass spectrum (ESI-IT, positive mode) of peptide CA-M (MW = 1769.2 Da), showing the quasi-molecular ion ([P+H]^+^), the di-protonated ([P+2H]^2+^, base peak), the tri-protonated ([P+3H]^3+^), and the tetra-protonated ([P+4H]^4+^) ions of the target peptide (P), Figure S6: RP-HPLC chromatogram for peptide CA-M after purification; gradient elution from 1 to 100% ACN in 0.05% aqueous TFA at 1 mL/min flow rate, for 30 min, on a C-18 column (150 × 4.6 mm ID and 5 µm pore size); detection at 220 nm, Figure S7: Mass spectrum (ESI-IT, positive mode) of peptide D4E1 (MW = 2079.4 Da), highlighting the di-protonated ([P+2H]^2+^, base peak), tri-protonated ([P+3H]^3+^) and tetra-protonated ([P+4H]^4+^) ions of the target peptide (P), Figure S8: RP-HPLC chromatogram for peptide D4E1, after purification; gradient elution from 1 to 100% ACN in 0.05% aqueous TFA at 1 mL/min flow rate, for 30 min, on a C-18 column (150 × 4.6 mm ID and 5 µm pore size); detection at 220 nm, Figure S9: Mass spectrum (ESI-IT, positive mode) of peptide 3.1 (MW = 1393.9 Da), showing the quasi-molecular ([P+H]^+^, base peak), di-protonated ([P+2H]^2+^), tri-protonated ([P+3H]^3+^) and tetra-protonated ([P+4H]^4+^) ions of the target peptide (P), Figure S10: RP-HPLC chromatogram for peptide 3.1, after purification; gradient elution from 1 to 100% ACN in 0.05% aqueous TFA at 1 mL/min flow rate, for 30 min, on a C-18 column (150 × 4.6 mm ID and 5 µm pore size); detection at 220 nm, Figure S11: Mass spectrum (ESI-IT, positive mode) of peptide Dhvar-5 (MW = 1845.3 Da), highlighting the di-protonated ion ([P+2H]^2+^, base peak), the tri-protonated ion ([P+3H]^3+^), the tetra-protonated ion ([P+4H]^4+^) and its sodium adduct ([P+4Na]^4+^), and the penta-sodium adduct ([P+5Na]^5+^) of the target peptide (P), Figure S12: RP-HPLC chromatogram for peptide Dhvar-5, after purification; gradient elution from 1 to 100% ACN in 0.05% aqueous TFA at 1 mL/min flow rate, for 30 min, on a C-18 column (150 × 4.6 mm ID and 5 µm pore size); detection at 220 nm, Figure S13: Growth curves of eight *E. amylovora* strains exposed to increasing concentrations of BP100 (0, 1.6, 3.4, and 5 µM). Vertical bars: mean value with standard deviation (*n* = 3), Figure S14: Growth curves of eight *E. amylovora* strains exposed to increasing concentrations of RW-BP100 (0, 1.6, and 3.4 µM). Vertical bars: mean value with standard deviation (*n* = 3), Figure S15: Growth curves of eight *E. amylovora* strains exposed to increasing concentrations of CA-M (0, 1.6, 3.4, and 5 µM). Vertical bars: mean value with standard deviation (*n* = 3), Table S1: AMPs antibiogram susceptibility. (+): inhibition occurred; (-): inhibition did not occur; C+: Type strain LMG 2024.
