# Supplementary material for: In Vitro Evaluation of Five Antimicrobial Peptides against the Plant Pathogen Erwinia amylovora
Source: Biomolecules. 2021 Apr 9;11(4):554. doi: 10.3390/biom11040554 (PMC8069920; doi:10.3390/biom11040554)
Supplement: Supplementary file 1 [file biomolecules-11-00554-s001.pdf]

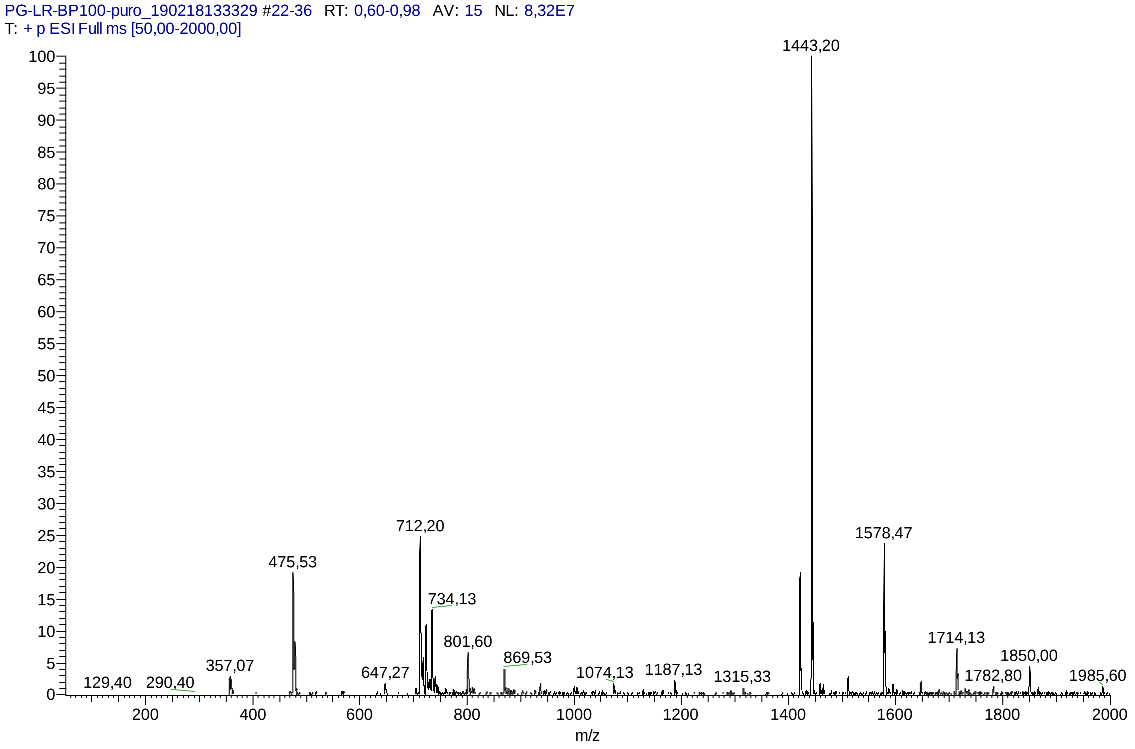

Figure S1. Mass spectrum (ESI-IT, positive mode) of peptide BP100 (MW=1419.9 Da), highlighting the quasi-molecular ion ($[P+H]^+$), its sodium adduct ($[P+Na]^+$, base peak), the di-protonated ($[P+2H]^{2+}$) ion and its sodium adduct ($[P+2Na]^{2+}$), and the tri-protonated ($[P+3H]^{3+}$) and tetra-protonated ($[P+4H]^{4+}$) ions of the target peptide (P).

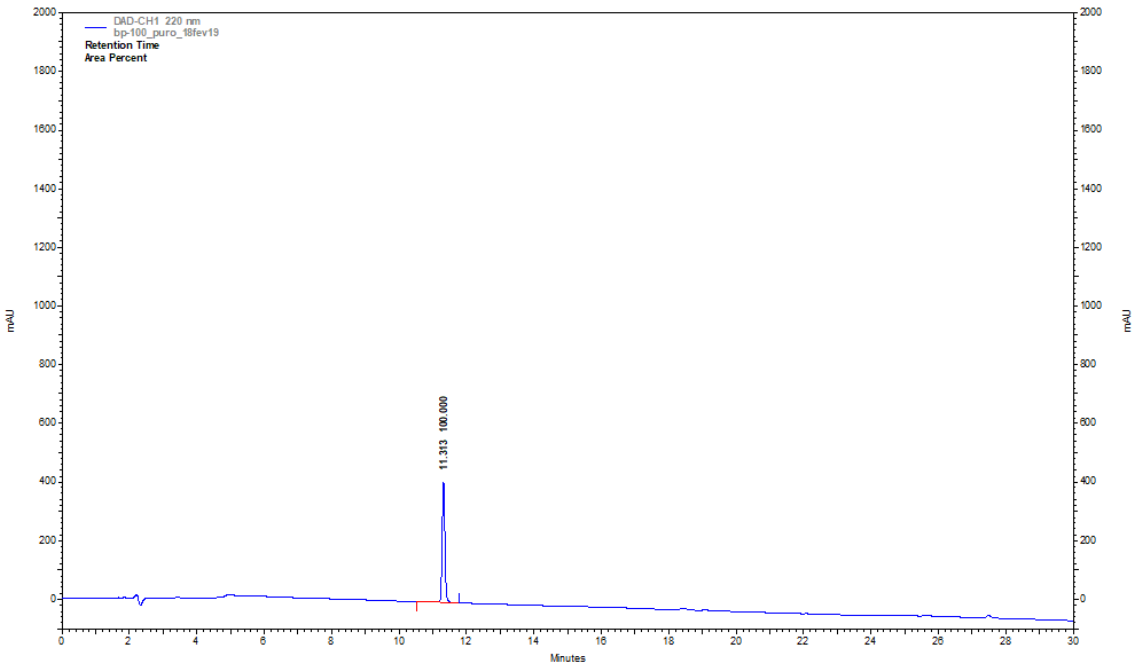

Figure S2. RP-HPLC chromatogram for peptide BP100, after purification; gradient elution from 1 to 100% ACN in 0.05% aqueous TFA at 1 mL/min flow rate, for 30 min, on a C-18 column (150 × 4.6 mm ID and 5 µm pore size); detection at 220 nm.

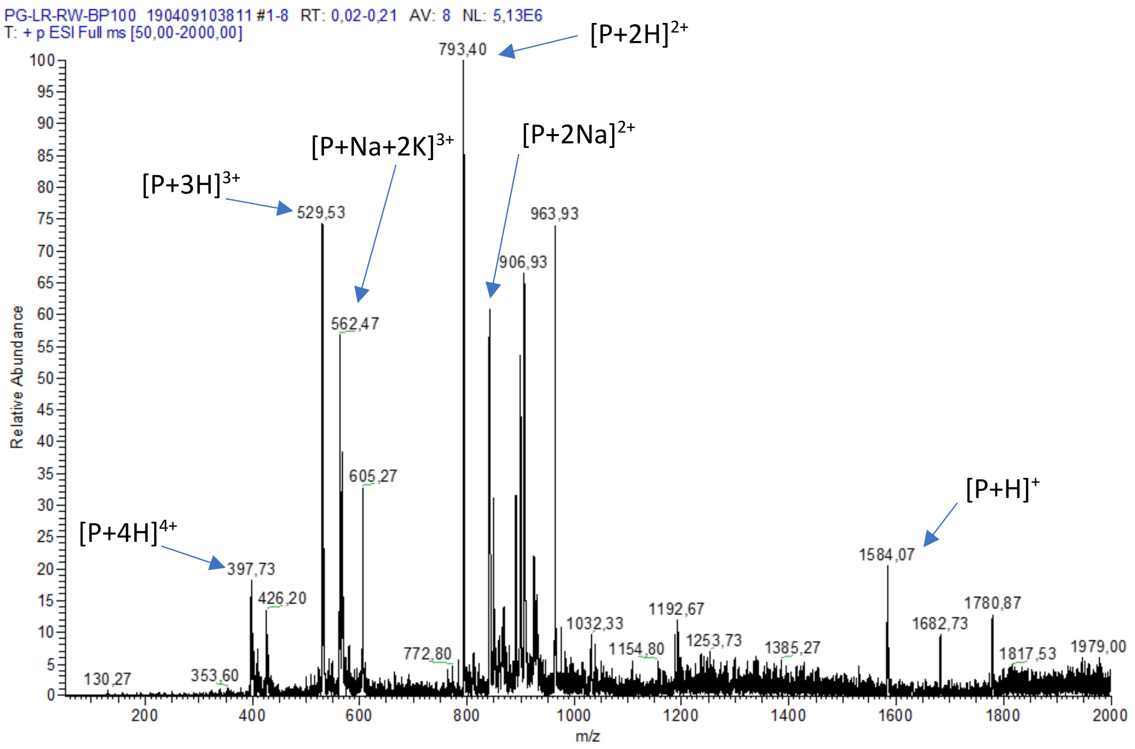

Figure S3. Mass spectrum (ESI-IT, positive mode) of peptide RW-BP100 (MW=1583.0 Da), highlighting the quasi-molecular ion ([P+H]$^+$), the di-protonated ([P+2H]$^{2+}$, base peak) ion and respective sodium adduct ([P+2Na]$^{2+}$), the tri-protonated ion ([P+3H]$^{3+}$) and its sodium and dipotassium adduct ([P+Na+2K]$^{3+}$), and the tetra-protonated ([P+4H]$^{4+}$) ion of the target peptide (P).

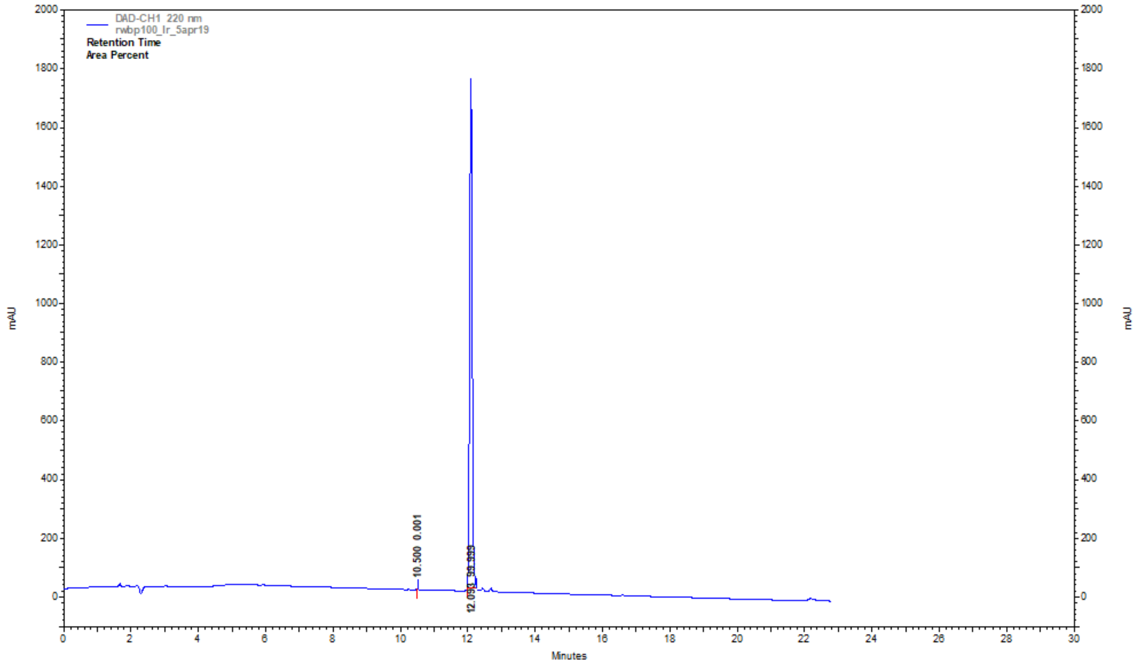

Figure S4. RP-HPLC chromatogram for peptide RW-BP100, after purification; gradient elution from 1 to 100% ACN in 0.05% aqueous TFA at 1 mL/min flow rate, for 30 min, on a C-18 column (150 × 4.6 mm ID and 5 µm pore size); detection at 220 nm.

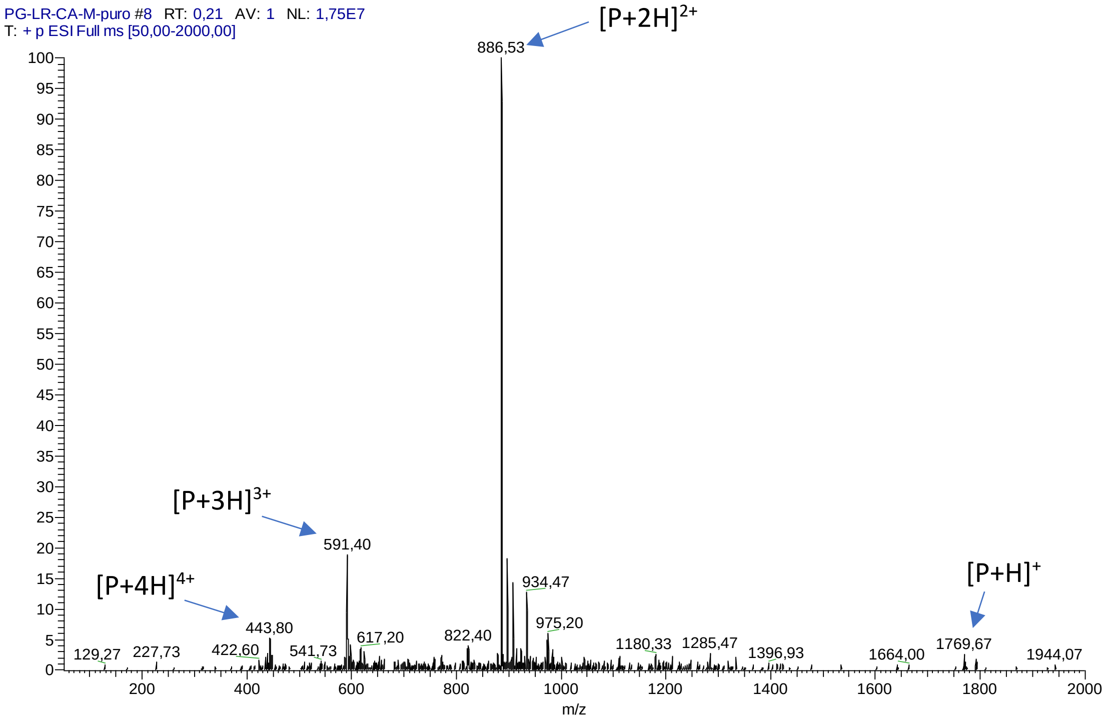

Figure S5. Mass spectrum (ESI-IT, positive mode) of peptide CA-M (MW=1769.2 Da), showing the quasi-molecular ion ($[P+H]^+$), the di-protonated ($[P+2H]^{2+}$, base peak), the tri-protonated ($[P+3H]^{3+}$), and the tetra-protonated ($[P+4H]^{4+}$) ions of the target peptide (P).

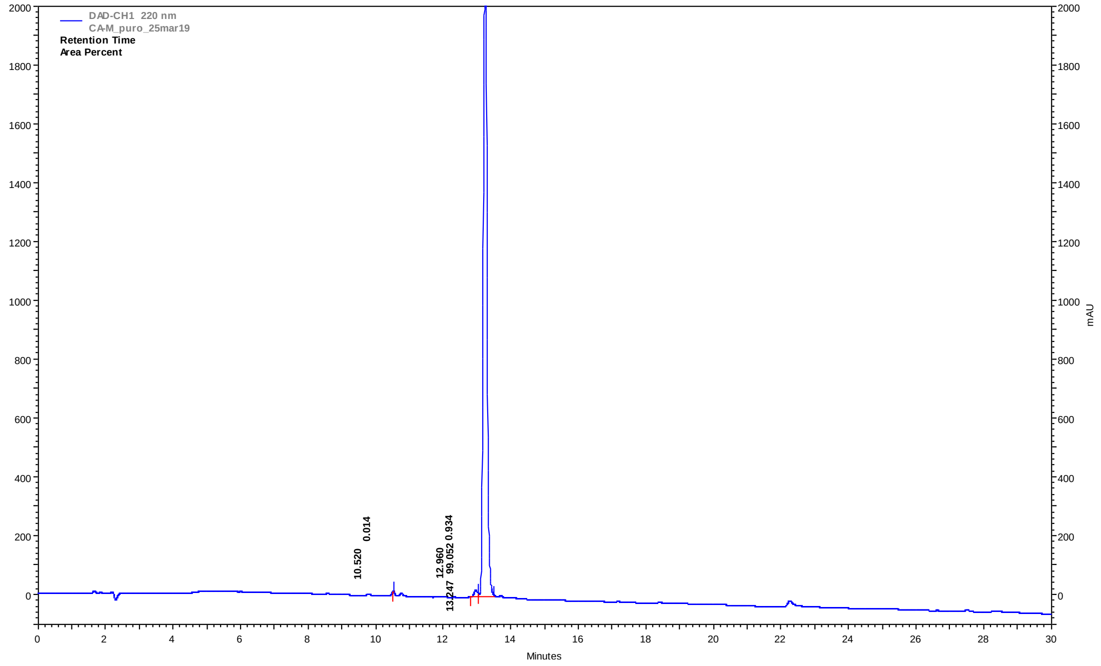

Figure S6. RP-HPLC chromatogram for peptide CA-M after purification; gradient elution from 1 to 100% ACN in 0.05% aqueous TFA at 1 mL/min flow rate, for 30 min, on a C-18 column (150 × 4.6 mm ID and 5 µm pore size); detection at 220 nm.

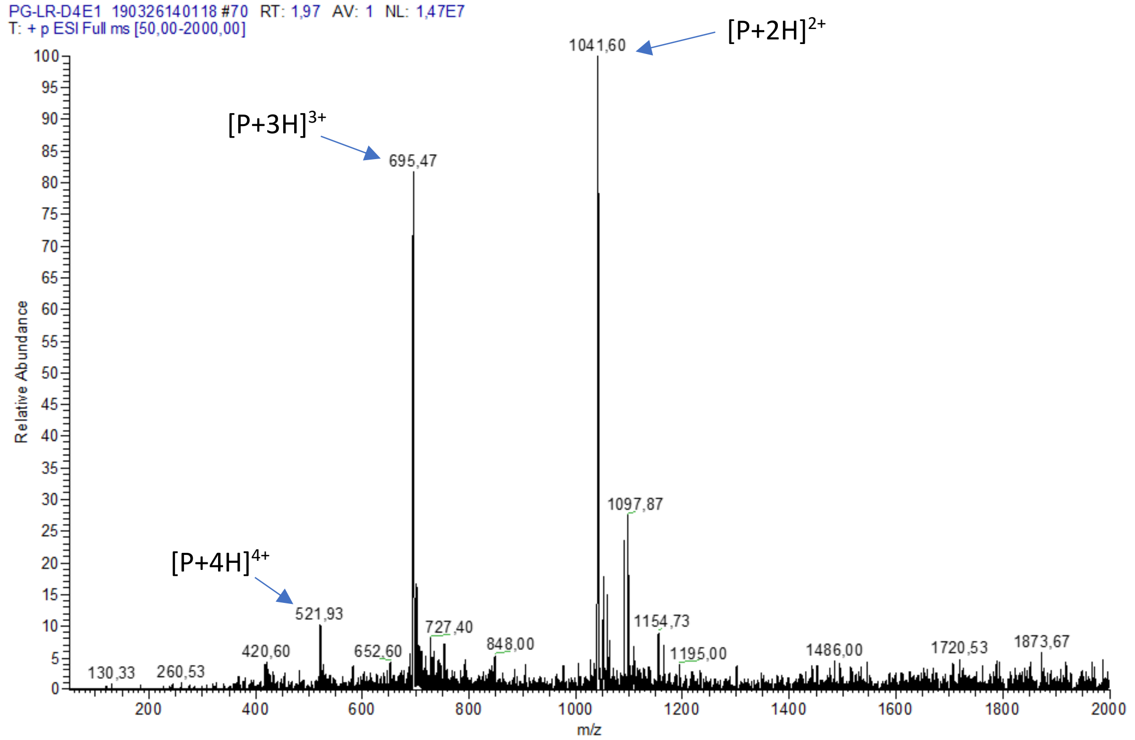

Figure S7. Mass spectrum (ESI-IT, positive mode) of peptide D4E1 (MW=2079.4 Da), highlighting the di-protonated ([P+2H]$^{2+}$, base peak), tri-protonated ([P+3H]$^{3+}$) and tetra-protonated ([P+4H]$^{4+}$) ions of the target peptide (P).

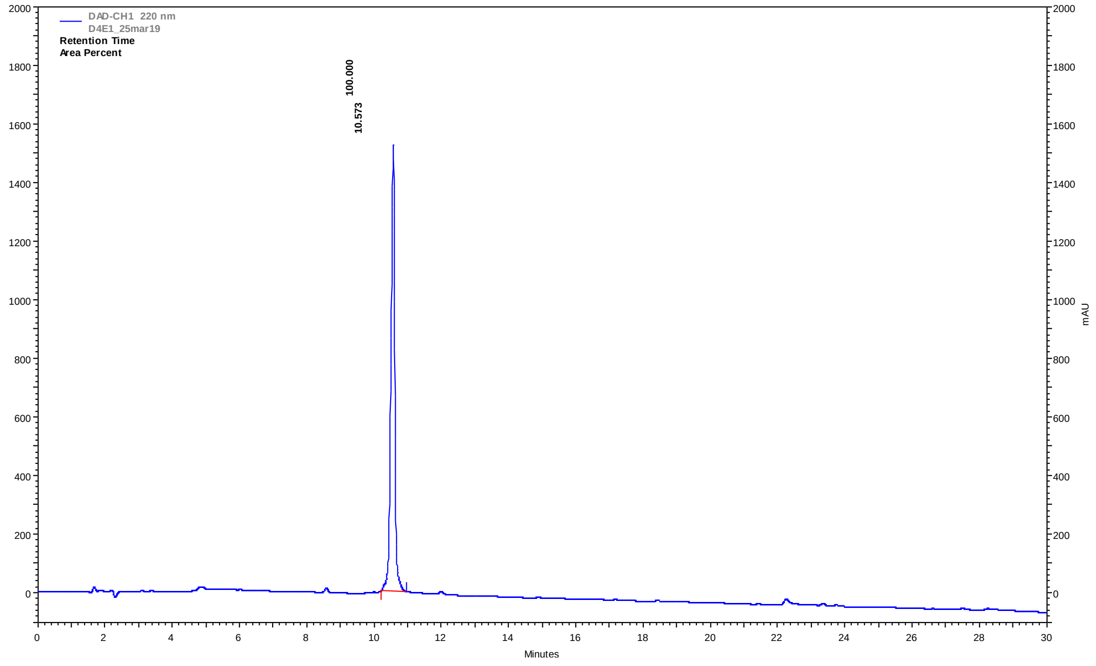

Figure S8. RP-HPLC chromatogram for peptide D4E1, after purification; gradient elution from 1 to 100% ACN in 0.05% aqueous TFA at 1 mL/min flow rate, for 30 min, on a C-18 column (150 × 4.6 mm ID and 5 µm pore size); detection at 220 nm.

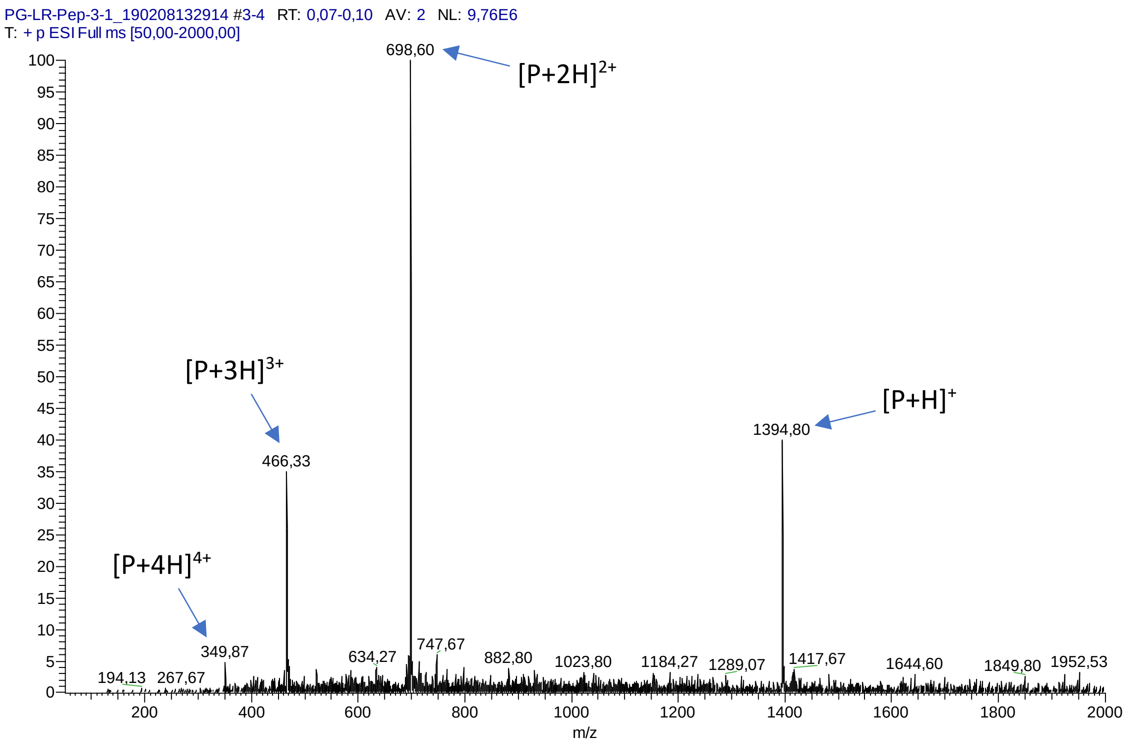

Figure S9. Mass spectrum (ESI-IT, positive mode) of peptide 3.1 (MW=1393.9 Da), showing the quasi-molecular ([P+H]$^{+}$, base peak), di-protonated ([P+2H]$^{2+}$), tri-protonated ([P+3H]$^{3+}$) and tetra-protonated ([P+4H]$^{4+}$) ions of the target peptide (P).

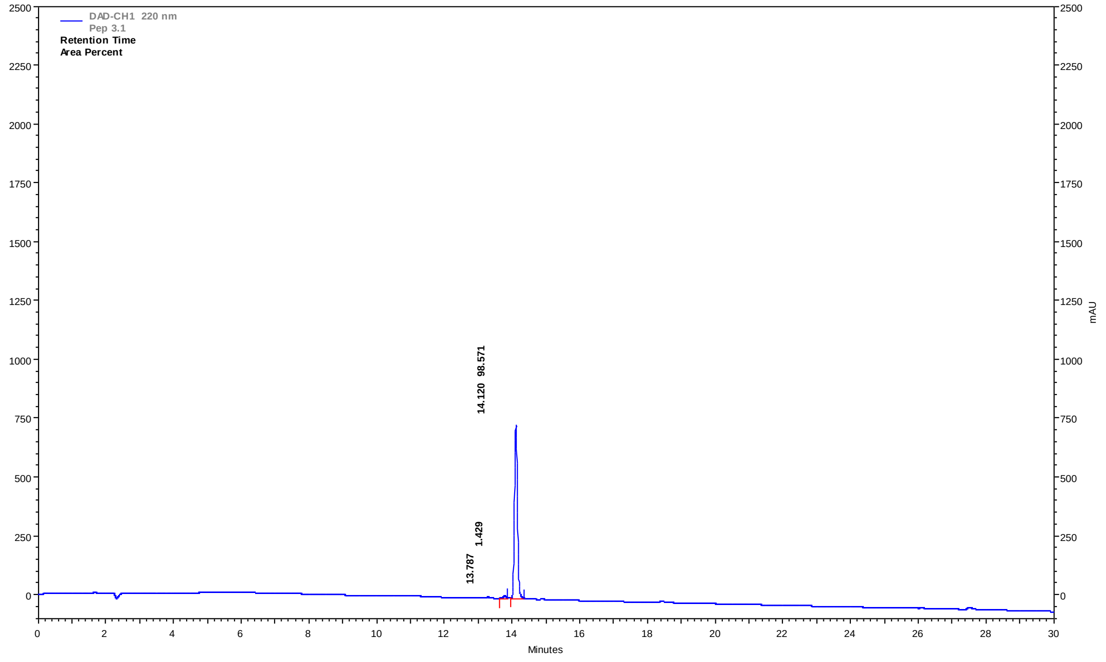

Figure S10. RP-HPLC chromatogram for peptide 3.1, after purification; gradient elution from 1 to 100% ACN in 0.05% aqueous TFA at 1 mL/min flow rate, for 30 min, on a C-18 column (150 × 4.6 mm ID and 5 µm pore size); detection at 220 nm.

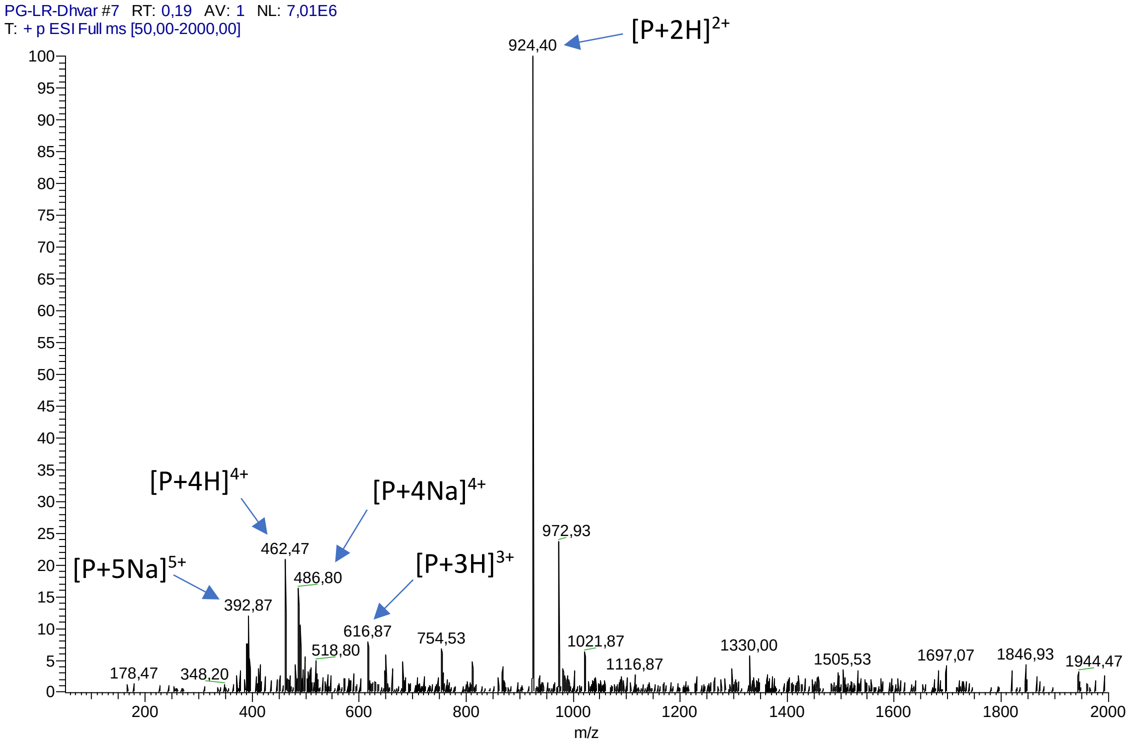

Figure S11. Mass spectrum (ESI-IT, positive mode) of peptide Dhvar-5 (MW=1845.3 Da), highlighting the di-protonated ion ([P+2H]$^{2+}$, base peak), the tri-protonated ion ([P+3H]$^{3+}$), the tetra-protonated ion ([P+4H]$^{4+}$) and its sodium adduct ([P+4Na]$^{4+}$), and the penta-sodium adduct ([P+5Na]$^{5+}$) of the target peptide (P).

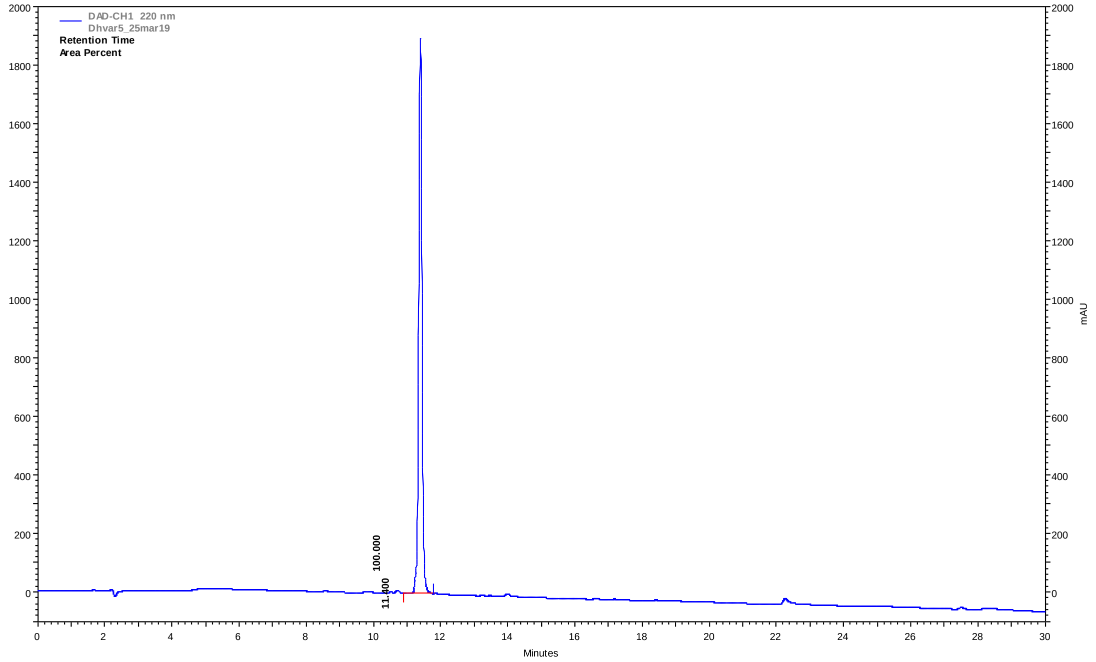

Figure S12. RP-HPLC chromatogram for peptide Dhvar-5, after purification; gradient elution from 1 to 100% ACN in 0.05% aqueous TFA at 1 mL/min flow rate, for 30 min, on a C-18 column (150 × 4.6 mm ID and 5 µm pore size); detection at 220 nm.

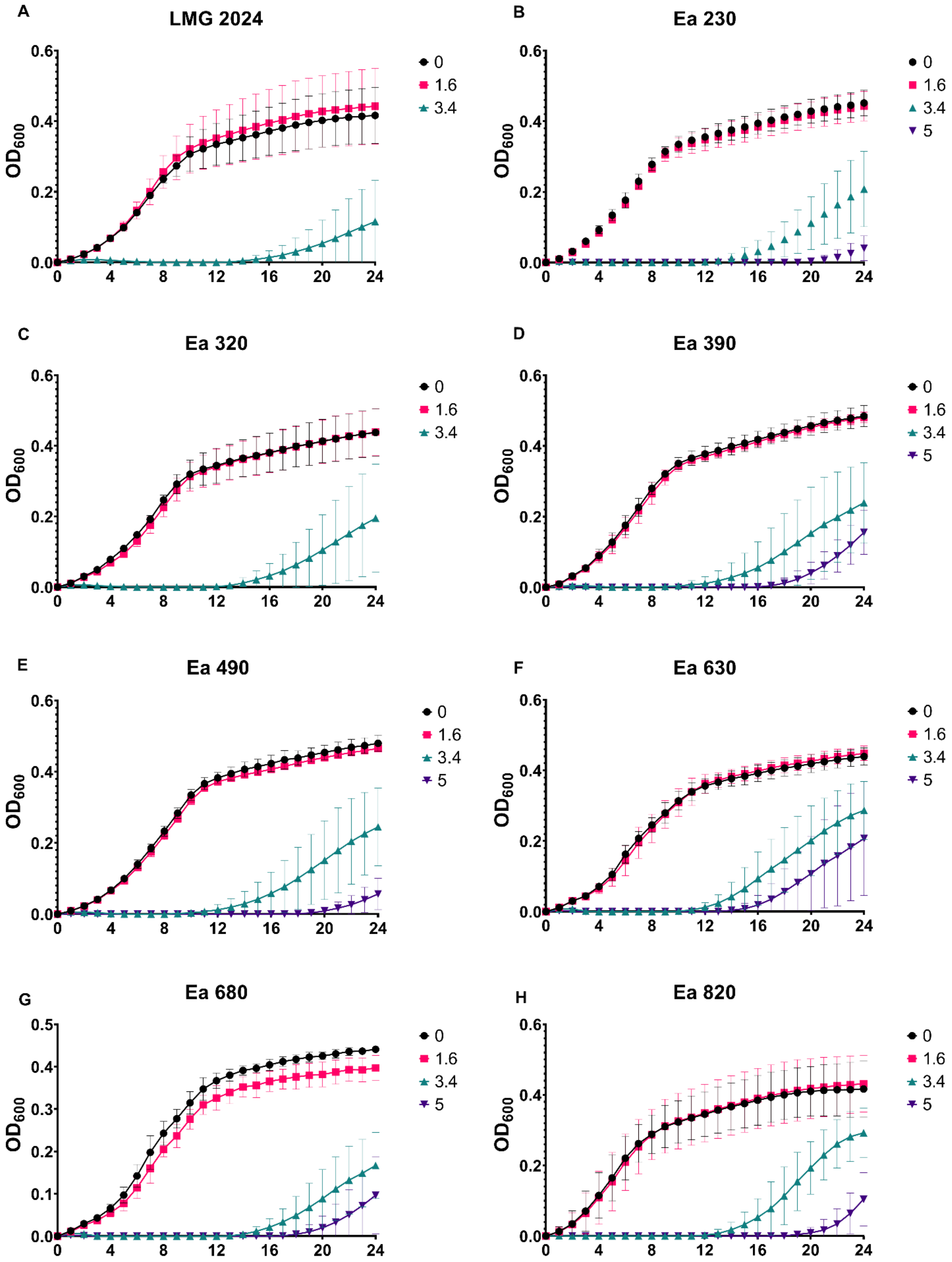

Figure S13. Growth curves of eight *E. amylovora* strains exposed to increasing concentrations of BP100 (0, 1.6, 3.4, and 5 µM). Vertical bars: mean value with standard deviation (n = 3).

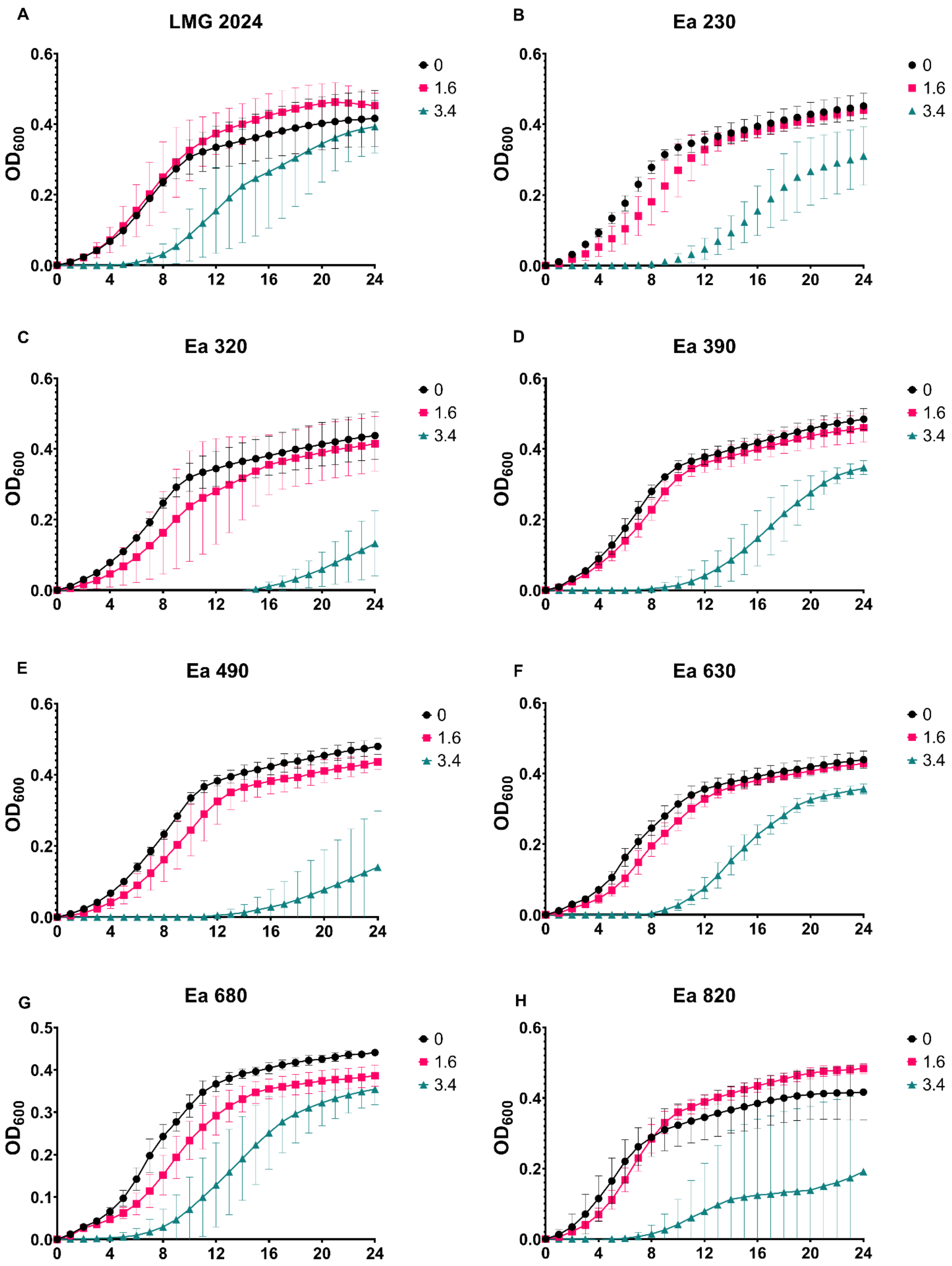

Figure S14. Growth curves of eight *E. amylovora* strains exposed to increasing concentrations of RW-BP100 (0, 1.6, and 3.4 µM). Vertical bars: mean value with standard deviation (n = 3).

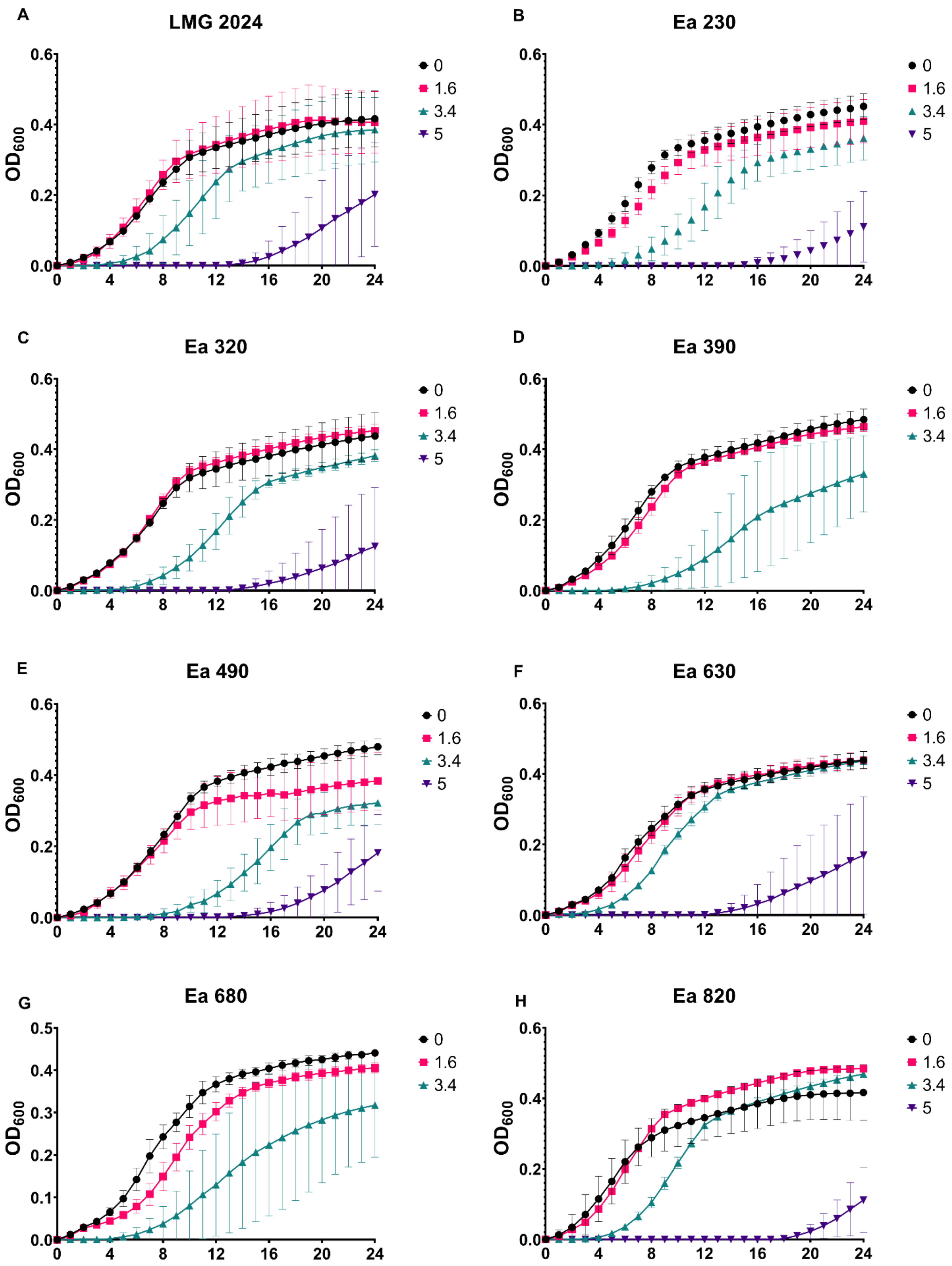

Figure S15. Growth curves of eight *E. amylovora* strains exposed to increasing concentrations of CA-M (0, 1.6, 3.4, and 5 µM). Vertical bars: mean value with standard deviation (n = 3).

Supplementary Table 1. AMPs antibiogram susceptibility. (+): inhibition occurred; (-): inhibition did not occur; C+: Type strain LMG 2024.

AMP	Strain	Concentrations (µM)							
**BP100**		0.4	1.6	6.2	25	50	100	150	200
	Ea 230	-	-	-	+	+	+	+	+
	Ea 240	-	-	-	+	+	+	+	+
	Ea 250	-	-	-	+	+	+	+	+
	Ea 260	-	-	-	+	+	+	+	+
	Ea 270	-	-	-	+	+	+	+	+
	Ea 280	-	-	-	+	+	+	+	+
	Ea 310	-	-	-	+	+	+	+	+
	Ea 320	-	-	-	+	+	+	+	+
	Ea 340	-	-	-	+	+	+	+	+
	Ea 350	-	-	-	+	+	+	+	+
	Ea 410	-	-	-	+	+	+	+	+
	Ea 430	-	-	-	+	+	+	+	+
	Ea 450	-	-	-	+	+	+	+	+
	Ea 460	-	-	-	+	+	+	+	+
	Ea 470	-	-	-	+	+	+	+	+
	Ea 480	-	-	-	+	+	+	+	+
	Ea 500	-	-	-	+	+	+	+	+
	Ea 510	-	-	-	+	+	+	+	+
	Ea 520	-	-	-	+	+	+	+	+
	Ea 540	-	-	-	+	+	+	+	+
	Ea 570	-	-	-	+	+	+	+	+
	Ea 580	-	-	-	+	+	+	+	+
	Ea 620	-	-	-	+	+	+	+	+
	Ea 630	-	-	-	+	+	+	+	+
	Ea 670	-	-	-	+	+	+	+	+
	Ea 720	-	-	-	+	+	+	+	+
	Ea 730	-	-	-	+	+	+	+	+
	Ea 740	-	-	-	+	+	+	+	+
	Ea 780	-	-	-	+	+	+	+	+
	Ea 790	-	-	-	+	+	+	+	+
	Ea 390	-	-	-	-	+	+	+	+
	Ea 490	-	-	-	-	+	+	+	+
	Ea 610	-	-	-	-	+	+	+	+
	Ea 680	-	-	-	-	+	+	+	+
	Ea 750	-	-	-	-	+	+	+	+
	Ea 820	-	-	-	-	+	+	+	+
	C+	-	-	-	-	+	+	+	+

AMP	Strain	Concentrations (µM)							
**RW-BP100**		0.4	1.6	6.2	25	50	100	150	200
	Ea 230	-	-	-	+	+	+	+	+
	Ea 240	-	-	-	+	+	+	+	+
	Ea 250	-	-	-	+	+	+	+	+
	Ea 260	-	-	-	+	+	+	+	+
	Ea 270	-	-	-	+	+	+	+	+
	Ea 280	-	-	-	+	+	+	+	+
	Ea 310	-	-	-	+	+	+	+	+
	Ea 320	-	-	-	+	+	+	+	+
	Ea 340	-	-	-	+	+	+	+	+
	Ea 350	-	-	-	+	+	+	+	+
	Ea 410	-	-	-	+	+	+	+	+
	Ea 430	-	-	-	+	+	+	+	+
	Ea 450	-	-	-	+	+	+	+	+
	Ea 460	-	-	-	+	+	+	+	+
	Ea 470	-	-	-	+	+	+	+	+
	Ea 480	-	-	-	+	+	+	+	+
	Ea 500	-	-	-	+	+	+	+	+
	Ea 510	-	-	-	+	+	+	+	+
	Ea 540	-	-	-	+	+	+	+	+
	Ea 620	-	-	-	+	+	+	+	+
	Ea 390	-	-	-	-	+	+	+	+
	Ea 490	-	-	-	-	+	+	+	+
	Ea 520	-	-	-	-	+	+	+	+
	Ea 570	-	-	-	-	+	+	+	+
	Ea 580	-	-	-	-	+	+	+	+
	Ea 610	-	-	-	-	+	+	+	+
	Ea 630	-	-	-	-	+	+	+	+
	Ea 670	-	-	-	-	+	+	+	+
	Ea 680	-	-	-	-	+	+	+	+
	Ea 720	-	-	-	-	+	+	+	+
	Ea 730	-	-	-	-	+	+	+	+
	Ea 740	-	-	-	-	+	+	+	+
	Ea 750	-	-	-	-	+	+	+	+
	Ea 790	-	-	-	-	+	+	+	+
	Ea 820	-	-	-	-	+	+	+	+
	Ea 780	-	-	-	-	-	+	+	+
	C+	-	-	-	-	-	+	+	+

AMP	Strain	Concentrations (μM)							
**CA-M**		0.4	1.6	6.2	25	50	100	150	200
	Ea 230	-	-	-	+	+	+	+	+
	Ea 240	-	-	-	+	+	+	+	+
	Ea 250	-	-	-	+	+	+	+	+
	Ea 260	-	-	-	+	+	+	+	+
	Ea 270	-	-	-	+	+	+	+	+
	Ea 280	-	-	-	+	+	+	+	+
	Ea 310	-	-	-	+	+	+	+	+
	Ea 320	-	-	-	+	+	+	+	+
	Ea 340	-	-	-	+	+	+	+	+
	Ea 350	-	-	-	+	+	+	+	+
	Ea 410	-	-	-	+	+	+	+	+
	Ea 430	-	-	-	+	+	+	+	+
	Ea 450	-	-	-	+	+	+	+	+
	Ea 460	-	-	-	+	+	+	+	+
	Ea 470	-	-	-	+	+	+	+	+
	Ea 480	-	-	-	+	+	+	+	+
	Ea 500	-	-	-	+	+	+	+	+
	Ea 510	-	-	-	+	+	+	+	+
	Ea 520	-	-	-	+	+	+	+	+
	Ea 540	-	-	-	+	+	+	+	+
	Ea 580	-	-	-	+	+	+	+	+
	Ea 620	-	-	-	+	+	+	+	+
	Ea 630	-	-	-	+	+	+	+	+
	Ea 670	-	-	-	+	+	+	+	+
	Ea 570	-	-	-	-	+	+	+	+
	Ea 610	-	-	-	-	+	+	+	+
	Ea 720	-	-	-	-	+	+	+	+
	Ea 740	-	-	-	-	+	+	+	+
	Ea 750	-	-	-	-	+	+	+	+
	Ea 780	-	-	-	-	+	+	+	+
	Ea 790	-	-	-	-	+	+	+	+
	Ea 820	-	-	-	-	+	+	+	+
	Ea 390	-	-	-	-	-	+	+	+
	Ea 490	-	-	-	-	-	+	+	+
	Ea 680	-	-	-	-	-	+	+	+
	Ea 730	-	-	-	-	-	+	+	+
	C+	-	-	-	-	-	+	+	+

AMP	Strain	Concentrations (μM)							
**3.1**		0.4	1.6	6.2	25	50	100	150	200
	Ea 230	-	-	-	-	+	+	+	+
	Ea 240	-	-	-	-	+	+	+	+
	Ea 250	-	-	-	-	+	+	+	+
	Ea 260	-	-	-	-	+	+	+	+
	Ea 270	-	-	-	-	+	+	+	+
	Ea 280	-	-	-	-	+	+	+	+
	Ea 310	-	-	-	-	+	+	+	+
	Ea 320	-	-	-	-	+	+	+	+
	Ea 430	-	-	-	-	+	+	+	+
	Ea 340	-	-	-	-	-	+	+	+
	Ea 350	-	-	-	-	-	+	+	+
	Ea 410	-	-	-	-	-	+	+	+
	Ea 450	-	-	-	-	-	+	+	+
	Ea 460	-	-	-	-	-	+	+	+
	Ea 470	-	-	-	-	-	+	+	+
	Ea 480	-	-	-	-	-	+	+	+
	Ea 490	-	-	-	-	-	+	+	+
	Ea 500	-	-	-	-	-	+	+	+
	Ea 510	-	-	-	-	-	+	+	+
	Ea 520	-	-	-	-	-	+	+	+
	Ea 540	-	-	-	-	-	+	+	+
	Ea 570	-	-	-	-	-	+	+	+
	Ea 580	-	-	-	-	-	+	+	+
	Ea 610	-	-	-	-	-	+	+	+
	Ea 620	-	-	-	-	-	+	+	+
	Ea 630	-	-	-	-	-	+	+	+
	Ea 670	-	-	-	-	-	+	+	+
	Ea 680	-	-	-	-	-	+	+	+
	Ea 720	-	-	-	-	-	+	+	+
	Ea 730	-	-	-	-	-	+	+	+
	Ea 740	-	-	-	-	-	+	+	+
	Ea 750	-	-	-	-	-	+	+	+
	Ea 780	-	-	-	-	-	+	+	+
	Ea 820	-	-	-	-	-	+	+	+
	Ea 790	-	-	-	-	-	-	+	+
	Ea 390	-	-	-	-	-	-	-	+
	C+	-	-	-	-	-	+	+	+

AMP	Strain	Concentrations (µM)							
**D4E1**		0.4	1.6	6.2	25	50	100	150	200
	Ea 280	-	-	-	+	+	+	+	+
	Ea 310	-	-	-	+	+	+	+	+
	Ea 320	-	-	-	+	+	+	+	+
	Ea 230	-	-	-	-	+	+	+	+
	Ea 240	-	-	-	-	+	+	+	+
	Ea 260	-	-	-	-	+	+	+	+
	Ea 270	-	-	-	-	+	+	+	+
	Ea 250	-	-	-	-	-	+	+	+
	Ea 430	-	-	-	-	-	+	+	+
	Ea 460	-	-	-	-	-	+	+	+
	Ea 470	-	-	-	-	-	+	+	+
	Ea 480	-	-	-	-	-	+	+	+
	Ea 490	-	-	-	-	-	+	+	+
	Ea 500	-	-	-	-	-	+	+	+
	Ea 510	-	-	-	-	-	+	+	+
	Ea 570	-	-	-	-	-	+	+	+
	Ea 610	-	-	-	-	-	+	+	+
	Ea 620	-	-	-	-	-	+	+	+
	Ea 630	-	-	-	-	-	+	+	+
	Ea 670	-	-	-	-	-	+	+	+
	Ea 680	-	-	-	-	-	+	+	+
	Ea 720	-	-	-	-	-	+	+	+
	Ea 730	-	-	-	-	-	+	+	+
	Ea 740	-	-	-	-	-	+	+	+
	Ea 750	-	-	-	-	-	+	+	+
	Ea 820	-	-	-	-	-	+	+	+
	Ea 340	-	-	-	-	-	-	+	+
	Ea 410	-	-	-	-	-	-	+	+
	Ea 450	-	-	-	-	-	-	+	+
	Ea 520	-	-	-	-	-	-	+	+
	Ea 540	-	-	-	-	-	-	+	+
	Ea 580	-	-	-	-	-	-	+	+
	Ea 790	-	-	-	-	-	-	+	+
	Ea 350	-	-	-	-	-	-	-	+
	Ea 390	-	-	-	-	-	-	-	+
	Ea 780	-	-	-	-	-	-	-	-
	C+	-	-	-	-	-	+	+	+

AMP	Strain	Concentrations (μM)							
**Dhvar-5**		0.4	1.6	6.2	25	50	100	150	200
	Ea 230	-	-	-	-	-	-	-	-
	Ea 240	-	-	-	-	-	-	-	-
	Ea 250	-	-	-	-	-	-	-	-
	Ea 260	-	-	-	-	-	-	-	-
	Ea 270	-	-	-	-	-	-	-	-
	Ea 280	-	-	-	-	-	-	-	-
	Ea 310	-	-	-	-	-	-	-	-
	Ea 320	-	-	-	-	-	-	-	-
	Ea 340	-	-	-	-	-	-	-	-
	Ea 350	-	-	-	-	-	-	-	-
	Ea 390	-	-	-	-	-	-	-	-
	Ea 410	-	-	-	-	-	-	-	-
	Ea 430	-	-	-	-	-	-	-	-
	Ea 450	-	-	-	-	-	-	-	-
	Ea 460	-	-	-	-	-	-	-	-
	Ea 470	-	-	-	-	-	-	-	-
	Ea 480	-	-	-	-	-	-	-	-
	Ea 490	-	-	-	-	-	-	-	-
	Ea 500	-	-	-	-	-	-	-	-
	Ea 510	-	-	-	-	-	-	-	-
	Ea 520	-	-	-	-	-	-	-	-
	Ea 540	-	-	-	-	-	-	-	-
	Ea 570	-	-	-	-	-	-	-	-
	Ea 580	-	-	-	-	-	-	-	-
	Ea 610	-	-	-	-	-	-	-	-
	Ea 620	-	-	-	-	-	-	-	-
	Ea 630	-	-	-	-	-	-	-	-
	Ea 670	-	-	-	-	-	-	-	-
	Ea 680	-	-	-	-	-	-	-	-
	Ea 720	-	-	-	-	-	-	-	-
	Ea 730	-	-	-	-	-	-	-	-
	Ea 740	-	-	-	-	-	-	-	-
	Ea 750	-	-	-	-	-	-	-	-
	Ea 780	-	-	-	-	-	-	-	-
	Ea 790	-	-	-	-	-	-	-	-
	Ea 820	-	-	-	-	-	-	-	-
	C+	-	-	-	-	-	-	-	-
